# Supplementary material for: The limits to parapatric speciation 3: evolution of strong reproductive isolation in presence of gene flow despite limited ecological differentiation
Source: Philos Trans R Soc Lond B Biol Sci. 2020 Jul 13;375(1806):20190532. doi: 10.1098/rstb.2019.0532 (PMC7423268; doi:10.1098/rstb.2019.0532)
Supplement: Supplementary information [file rstb20190532supp3.pdf]

SI-The limits to parapatric speciation 3: Evolution of strong reproductive isolation in presence of gene flow despite limited ecological differentiation

Alexandre Blanckaert *[1,2], Claudia Bank[2], and Joachim Hermisson[1,3]

[1]Department of Mathematics, University of Vienna, 1090 Vienna, Austria

[2]Instituto Gulbenkian de Ciência, 2780-156 Oeiras, Portugal

[3]Mathematics and Biosciences Group, Max F. Perutz Laboratories, 1030 Vienna, Austria

April 14, 2020

S 1 Part I: the two and three-locus models

S 1.1 Model

The model for three loci was introduced in the main manuscript. Here, additional information and details regarding the 3-locus model are presented. In particular, we expand equation (1) and provide the system of equations for the three diallelic locus model with a detailed expression both for the fitness term w_X and the recombination function $f_R(X)$ (eq. (S1) and (S2)). If C appears on the island, $m_C = 0$, otherwise $m_C = m$. $\bar{w}$ corresponds to the mean fitness of the population and is given by $\bar{w} = \alpha x_2 + \beta x_3 + \gamma x_4 + (\alpha + \beta + \epsilon_{AB})x_5 + (\alpha + \gamma + \epsilon_{AC})x_6 + (\beta + \gamma + \epsilon_{BC})x_7 + (\alpha + \beta + \gamma + \epsilon_{AB} + \epsilon_{BC} + \epsilon_{AC} + \epsilon_{ABC})x_8$.

*blanckaert.a@gmail.com

$$
\begin{cases}
\dot{x_1} = & x_1(0-\bar{w}-m) - r_{AB}D_{12,A} - r_{BC}D_{14,C} \\
\dot{x_2} = & x_2(\alpha-\bar{w}-m) - r_{AB}D_{21,a} - r_{BC}D_{26,C} \\
\dot{x_3} = & x_3(\beta-\bar{w}-m) - r_{AB}D_{35,A} - r_{BC}D_{37,C} + m - m_C \\
\dot{x_4} = & x_4(\gamma-\bar{w}-m) - r_{AB}D_{46,A} - r_{BC}D_{41,c} \\
\dot{x_5} = & x_5(\alpha+\beta+\epsilon_{AB}-\bar{w}-m) - r_{AB}D_{53,a} - r_{BC}D_{58,C} \\
\dot{x_6} = & x_6(\alpha+\gamma+\epsilon_{AC}-\bar{w}-m) - r_{AB}D_{64,a} - r_{BC}D_{62,c} \\
\dot{x_7} = & x_7(\beta+\gamma+\epsilon_{BC}-\bar{w}-m) - r_{AB}D_{78,A} - r_{BC}D_{73,c} + m_C \\
\dot{x_8} = & x_8(\alpha+\beta+\gamma+\epsilon_{AB}+\epsilon_{BC}+\epsilon_{BC}+\epsilon_{ABC}-\bar{w}-m) - r_{AB}D_{87,a} - r_{BC}D_{85,c}
\end{cases}
\tag{S1}
$$

$$
\text{with}
\begin{cases}
D_{ij,X} = x_i p_X - x_j(1-p_X) \\
p_A = (1-p_a) = x_2 + x_5 + x_6 + x_8 \\
p_B = (1-p_b) = x_3 + x_5 + x_7 + x_8 \\
p_C = (1-p_c) = x_4 + x_6 + x_7 + x_8
\end{cases}
\tag{S2}
$$

We assume that the loci A, B and C are in this order along the chromosome. The recombination rate between loci A and B is given by r_{AB} and between B and C by r_{BC}. In addition, we provide a decomposition of the system of equations, using $\dot{x_2}$ as an example and emphasizing which term corresponds to which biological mechanism, equation (S3).

$$
\begin{aligned}
\dot{x_2} = \quad & \underbrace{x_2(\alpha-\bar{w})}_{\text{(soft) selection}} - \underbrace{x_2 * m}_{\text{individuals replaced by migrants}} \\
& \underbrace{-\,r_{AB}\Big(\underbrace{x_2(1-p_A)}_{\text{destruction of haplotype } Abc} - \underbrace{x_1 p_A)}_{\text{formation of haplotype } Abc} \Big)}_{\text{recombination between the loci } A \text{ and } B} \\
& \underbrace{-\,r_{BC}\Big(\underbrace{x_2 p_C}_{\text{destruction of haplotype } Abc} - \underbrace{x_6(1-p_C)}_{\text{formation of haplotype } Abc} \Big)}_{\text{recombination between the loci } B \text{ and } C}
\end{aligned}
\tag{S3}
$$

For loose linkage, the system of equations given in eq. (S1) reduces to equation (S4).

$$
\begin{cases}
\dot{p_A} = & p_A(\alpha(1-p_B)(1-p_C) + (\alpha+\beta+\epsilon_{AB})p_B(1-p_C) + (\alpha+\gamma+\epsilon_{ac})(1-p_B)p_C \\
& +(\alpha+\beta+\gamma+\epsilon_{AB}+\epsilon_{ac}+\epsilon_{bc}+\epsilon_{abc})p_Bp_C - \bar{w} - m) \\
\dot{p_B} = & p_B(\beta(1-p_A)(1-p_C) + (\alpha+\beta+\epsilon_{AB})p_A(1-p_C) + (\beta+\gamma+\epsilon_{bc})(1-p_A)p_C \\
& +(\alpha+\beta+\gamma+\epsilon_{AB}+\epsilon_{ac}+\epsilon_{bc}+\epsilon_{abc})p_Ap_C - \bar{w} - m) + m \\
\dot{p_C} = & p_C(\gamma(1-p_A)(1-p_B) + (\alpha+\gamma+\epsilon_{ac})p_A(1-p_B) + (\beta+\gamma+\epsilon_{bc})(1-p_A)p_B \\
& +(\alpha+\beta+\gamma+\epsilon_{AB}+\epsilon_{ac}+\epsilon_{bc}+\epsilon_{abc})p_Ap_B - \bar{w} - m) + m_C
\end{cases}
\tag{S4}
$$

S 1.2 2-locus model

For the two locus model in loose linkage, the expression for $m^{Ab}_{\max}$ has been previously derived (see Bank et al. [2012], equation 11) for negative epistasis($\epsilon_{AB} < 0$). Below, we provide this expression using our notation:

$$
\begin{cases}
-\min(\alpha/2, \alpha+\beta) \leq \epsilon_{AB} \leq \min(0, -\beta) & m^{Ab}_{\max} = \frac{-(\alpha+\epsilon_{AB})(\beta+\epsilon_{AB})}{\alpha} \\
\beta \leq \epsilon_{AB} < -(\alpha+\beta) & m^{Ab}_{\max} = \frac{\alpha\beta}{\beta+\epsilon_{AB}} \\
\epsilon_{AB} < -\max(\alpha/2, |\beta|) & m^{Ab}_{\max} = -\frac{(\alpha+\beta)(\beta+\epsilon_{AB})}{\alpha}.
\end{cases}
\tag{S5}
$$

S 1.2.1 Extension to positive epistasis

We now consider two epistatically interacting loci, A and B in loose linkage. All results for maximal migration rates can be derived explicitly (for negative epistasis, see Bank et al. [2012].) For a more complete analysis, we extend the definition of $m^{Ab}_{\max}$ to positive epistasis between A and B:

$$
\begin{cases}
\epsilon_{AB} > 0 \text{ and } \alpha+\beta+\epsilon_{AB} < 0 & m^{Ab}_{\max} = \frac{\alpha\beta}{\beta+\epsilon_{AB}} \\
\epsilon_{AB} > 0 \text{ and } \alpha+\beta+\epsilon_{AB} > 0 \text{ and } \beta+\epsilon_{AB} < 0 & m^{Ab}_{\max} = -\frac{(\alpha+\beta)(\beta+\epsilon_{AB})}{\alpha}
\end{cases}
\tag{S6}
$$

The inclusion of positive epistasis modifies the definition of local adaptation $\Lambda^{Ab}_{\max} = \text{Max}[\alpha-\beta, \alpha, \alpha+\epsilon_{AB}]$. However, if AB is the best haplotype on the island, there is no 2-locus genetic barrier. Therefore, $w(Ab) > w(AB)$ is a necessary condition for the existence of a genetic barrier, leading to $\beta + \epsilon_{AB} < 0$. Given that $\epsilon_{AB} > 0$, we can deduce that $\beta < 0$ and $-\beta > \epsilon_{AB}$. This means that we can safely ignore the third term in the definition of $\Lambda^{Ab}_{\max}$. Under these conditions, the definition of the maximum amount of local adaptation in the 2-locus model remains unchanged.

We have established in the main manuscript that $m^{Ab}_{\max} \leq \Lambda^{Ab}_{\max}$, independently of the sign of epistasis. If we compare the 2-locus barrier to its equivalent without epistasis, the genetic barrier is strengthened whenever there is some asymmetry in the selection strength at both loci ($\alpha \neq -\beta$) and when there is epistasis between the two derived alleles, ϵ_{AB}, according to the following conditions:

$$\begin{cases} -(\alpha+\beta) \leq \epsilon_{AB} \leq \mathrm{Max}[0,-\beta] \text{ if } \beta \leq \dfrac{-\alpha}{2} \\ -\dfrac{\alpha\beta}{\alpha+4\beta} \leq \epsilon_{AB} \leq \mathrm{Max}[0,-\beta] \text{ if } \beta > \dfrac{-\alpha}{2} \\ 0 < \epsilon_{AB} < \dfrac{1}{2}(-\alpha-\beta+\sqrt{(\alpha+\beta)(\beta-3\alpha)}) \text{ if } 0 < \alpha < -\beta \end{cases} \tag{S7}$$

From equation (S7), we can deduce that if selection against the continental allele B is weaker than selection for allele A ($-\beta < \alpha$), then allele B can become fixed at a lower migration rate than allele A. Negative epistasis can strengthen the genetic barrier (in comparison to the case without epistasis). Vice-versa, if $\alpha < -\beta$, A is lost first, and positive epistasis can strengthen the genetic barrier (in comparison to the case without epistasis), as illustrated in Figure S1. In the former case ($\alpha > -\beta$), A and B form a DMI ($\epsilon_{AB} < 0$) and we obtain the same upper bound $m^{Ab}_{\max} \leq \frac{\Lambda^{Ab}_{\max}}{2}$ as in the case with non-interacting loci. For $-\beta > \alpha$, the maximal barrier strength can exceed the threshold for non-interacting loci and reaches $m^{Ab}_{\max} = \Lambda^{Ab}_{\max}$ in the limit $\alpha \to 0$, $\beta \to -\Lambda^{Ab}_{\max}$ and $\epsilon_{AB} \to \Lambda^{Ab}_{\max}$.

For a given (α, β), we can estimate the strength of epistasis for which the genetic barrier is maximal. This condition is given in equation (S8) and the expression for the corresponding strength of the genetic barrier is provided in the main manuscript, equation (2).

$$\epsilon_{AB} = \begin{cases} -\frac{\alpha+\beta}{2} & \text{if } \alpha+\beta > 0 \text{ and } \beta \leq 0 \\ -(\alpha+\beta) & \text{if } \alpha+\beta < 0 \\ -\infty & \text{if } \beta > 0 \end{cases} \tag{S8}$$

S 1.2.2 Strongest genetic barrier for a given value of $\Lambda^{Ab}_{\max}$

One can wonder when the strongest barrier is formed for a given value of $\Lambda^{Ab}_{\max}$. From the previous paragraph, if we only allow for negative epistasis, the absence of epistasis and a fair repartition of local adaptation is the best solution.

If we now assume that epistasis is positive, then $\beta < 0$ is a necessary condition for the existence of the 2-locus genetic barrier, leading to $\Lambda^{Ab}_{\max} = \alpha - \beta$. Then, assuming $\alpha = \Lambda^{Ab}_{\max} + \beta$ and using equation (S6), one can infer when $m^{Ab}_{\max}$ is maximized. If $\alpha + \beta + \epsilon_{AB} < 0$, then $m^{Ab}_{\max}$

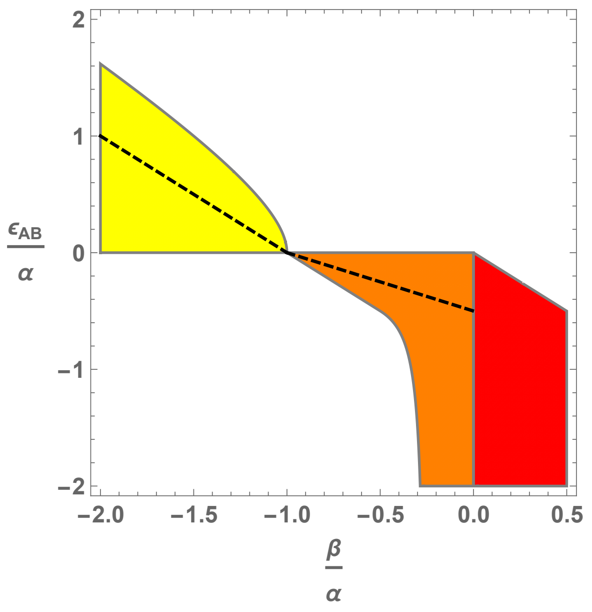

Figure S1: **Range of parameters where the presence of epistasis increases the strength of the genetic barrier $m^{Ab}_{\mathbf{max}}$.** The X axis corresponds to the selective advantage of allele B on the island and the Y axis corresponds to the strength of epistasis between A and B. The filled area indicates that epistasis between A and B leads to a stronger genetic barrier than the barrier formed if A and B do not interact. More precisely, yellow indicates that allele B can rescue allele A and orange that allele A can prevent the fixation of allele B. Red indicates that the genetic barrier only exists in the presence of epistasis. It is an illustration of equation (S7). The black dashed line indicates the strongest genetic barrier for a given $\{\alpha, \beta\}$. Its expression is given in equation (S8).

is an increasing function of ϵ_{AB} and reaches its maximum for $\epsilon_{AB} = -\Lambda^{Ab}_{\max} - 2\beta$, leading to $m^{Ab}_{\max} \leq -\beta$. If $\alpha + \beta + \epsilon_{AB} > 0$, then $m^{Ab}_{\max}$ is a decreasing function of ϵ_{AB} on $[-\frac{\Lambda^{Ab}_{\max}+2\beta}{2}, \infty[$. Alternatively, $m^{Ab}_{\max}$ is defined on $[\mathrm{Max}[0, (-\Lambda^{Ab}_{\max}+2\beta)], -\beta]$ and therefore reaches its maximum for $\epsilon_{AB} = -\Lambda^{Ab}_{\max} - 2\beta$, leading to $m^{Ab}_{\max} \leq -\beta$.

The optimal choice of parameters in this case corresponds to $\alpha \to 0$, $\beta \to -\Lambda^{Ab}_{\max}$ and $\epsilon_{AB} \to \Lambda^{Ab}_{\max}$. Figure S2 illustrates the dynamics of the system under these conditions ($\alpha = 0$, $\beta = -\Lambda^{Ab}_{\max}$ and $\epsilon_{AB} = \Lambda^{Ab}_{\max}$). With this parameter combination, there is no single point equilibrium. The solution is an isocline to which all trajectories converge.

This section technically covers all cases, including the case of two island or two continental adaptations since the system is fully parameterized with 2 selective advantage parameters and one epistatic term. Nevertheless, we will detail these two cases in the following sections.

S 1.2.3 Epistasis between two island adaptations

We now consider two mutations A_i and A_j appearing on the island, therefore $\alpha_i > 0$ and $\alpha_j > 0$. If A_i and A_j are in tight linkage, then $m^{A_iA_j}_{\max} = \alpha_i + \alpha_j + \epsilon_{A_iA_j}$ if $\epsilon_{A_iA_j} \leq \mathrm{Max}[-\alpha_i, -\alpha_j]$.

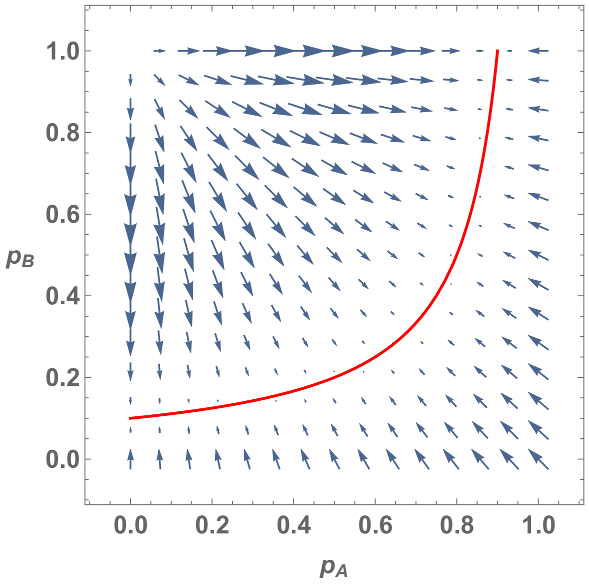

Figure S2: **Dynamics of the 2-locus model if $\alpha = 0$ and $\epsilon_{AB} = -\beta = \Lambda^{Ab}_{\mathbf{max}}$.** The X axis corresponds to the frequency of allele A and the Y axis to the frequency allele B. The red line corresponds to both p_A and p_B isoclines. The blue arrows indicate potential trajectories. Here, we use $m = 0.1\Lambda^{Ab}_{\max}$.

$m^{A_iA_j}_{\max}$=$\Lambda^{A_iA_j}_{\max}$ is always true in this setting. We now assume that A_i and A_j are in loose linkage. The amount of local adaptation is then given by $\Lambda^{A_iA_j}_{\max} = \alpha_i + \alpha_j + \epsilon_{A_iA_j}$. Indeed, $\Lambda^{A_iA_j}_{\max} = \alpha_i$ (or α_j) is not compatible with the existence of the 2-locus genetic barrier. By applying the transformation given in Table S1, we obtain that the genetic barrier will reach $\Lambda_{\max}$ for $\alpha_i = \Lambda^{A_iA_j}_{\max}, \alpha_j = \Lambda^{A_iA_j}_{\max}$ and $\epsilon_{A_iA_j} \to -\Lambda^{A_iA_j}_{\max}$. This corresponds to maximizing the selective advantage of both alleles A_i and A_j when they are rare, making them more resistant to swamping.

1st option	a_ia_j	A_ia_j	a_iA_j	A_iA_j
	0	α_i	α_j	$\alpha_i + \alpha_j + \epsilon_{A_iA_j}$
2nd option	a_kB_l	a_kb_l	A_kB_l	A_kb_l
	0	$-\beta_l$	$\alpha_k + \epsilon_{A_kB_l}$	$\alpha_k - \beta_l$

Table S1: **Fitness table and equivalence between two island adaptations A_i and A_j interacting with each other, and an island adaptation A_k interacting with a continental adaptation B_l.** Here, the continental haplotype has a fitness of zero on the island. Then, the two parametrizations are equivalent with the following change: $\alpha_i = -\beta_l, \alpha_j = \alpha_k + \epsilon_{A_kB_l}$ and $\epsilon_{A_iA_j} = -\epsilon_{A_kB_l}$.

Figure S3 illustrates the parameter space for which epistasis can strengthen a genetic barrier by A_i and A_j (in comparison with the case without epistasis), while maintaining the amount of local adaptation constant. Epistasis will strengthen the genetic barrier if it is negative, therefore allowing every allele to have a higher marginal fitness when rare, relative to the case without

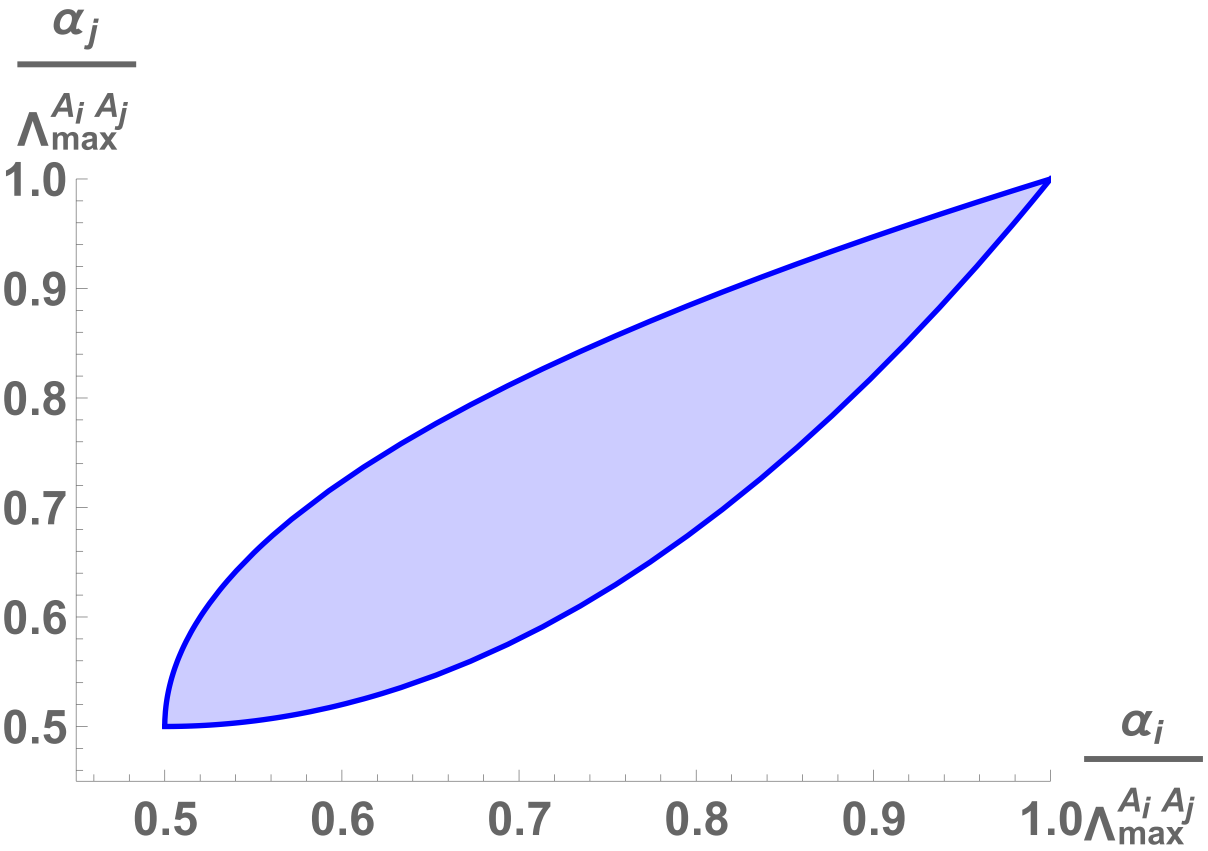

Figure S3: **Range of parameters for which epistasis between 2 island adaptations improves the genetic barrier, for a fixed amount of local adaptation.** The X axis corresponds to the selective advantage of allele A_i and the Y axis to the selective advantage of A_j. The blue area corresponds to parameters for which having epistasis between A_i and A_j generates a stronger genetic barrier compared to the absence of epistasis. The amount of local adaptation is identical for any combination of α_i and α_j.

epistasis.

Without loss of generality, we can assume $\Lambda_{\max}^{A_iA_j} = 1$. We then define $\epsilon_{A_iA_j} = 1 - \alpha_i - \alpha_j$, leading to the following expression for the strength of the 2-locus genetic barrier:

$$
\begin{aligned}
m_{\max}^{A_iA_j} &= \tfrac{(-1+\alpha_j)\alpha_j}{-1+\alpha_i} \\
\text{if} \quad & \alpha_i < 1 \text{ and } ((\alpha_i \geq \alpha_j \text{ and } 2\alpha_i + \alpha_j \geq 1 \text{ and } \alpha_i + \alpha_j \leq 1) \text{ or } (\alpha_i > \alpha_j \text{ and } \alpha_i + \alpha_j > 1)) \\
m_{\max}^{A_iA_j} &= \tfrac{(-1+\alpha_i)\alpha_i}{-1+\alpha_j} \\
\text{if} \quad & \alpha_j < 1 \text{ and } ((\alpha_i + \alpha_j > 1 \text{ and } \alpha_i < \alpha_j) \text{ or } (\alpha_i + 2\alpha_j \geq 1 \text{ and } \alpha_i \leq \alpha_j \text{ and } \alpha_i + \alpha_j \leq 1)) \\
m_{\max}^{A_iA_j} &= \tfrac{-(-1+\alpha_i)(-1+\alpha_j)}{4(-1+\alpha_i+\alpha_j)} \\
\text{if} \quad & \alpha_i + \alpha_j \leq 1 \text{ and } ((\alpha_i + 2\alpha_j < 1 \text{ and } ((\alpha_i < \alpha_j \text{ and } 2\alpha_i + \alpha_j \leq 1) \text{ or } 2\alpha_i + \alpha_j < 1)) \\
& \text{ or } (\alpha_i > \alpha_j \text{ and } 2\alpha_i + \alpha_j < 1))
\end{aligned}
\tag{S9}
$$

Because A_i and A_j are equivalent, we only need to determine the maximum of $m_{\max}^{A_iA_j}$ for the first and third terms of equation (S9).

First we consider $m_{\max}^{A_iA_j} = \frac{-(-1+\alpha_i)(-1+\alpha_j)}{4(-1+\alpha_i+\alpha_j)}$. $m_{\max}^{A_iA_j}$ is an increasing function of α_i if $0 < \alpha_j < 1$ (since its derivative with respect to α_i is $\frac{dm_{\max}^{A_iA_j}}{d\alpha_i} = \frac{-\alpha_j(-1+\alpha_j)}{4(-1+\alpha_i+\alpha_j)^2}$). Per symmetry, $m_{\max}^{A_iA_j}$ is an increasing function of α_j if $0 < \alpha_i < 1$. In addition, for this specific expression of the genetic barrier (third term), $\alpha_i + \alpha_j \leq 1$ is a necessary condition. Therefore, for a given $\alpha_i \in]0, 1[$, the strongest genetic barrier is obtained for $\alpha_j = 1 - \alpha_i$, implying $\epsilon_{A_iA_j} = 0$, leading to $\alpha_i = \alpha_j = \frac{1}{2}$.

We now consider $m_{\max}^{A_iA_j} = \frac{(-1+\alpha_j)\alpha_j}{-1+\alpha_i}$. Since $\alpha_i < 1$, $m_{\max}^{A_iA_j}$ is an increasing function of $\alpha_j \in [0, .5]$ and a decreasing function of $\alpha_j \in [.5, 1]$. In addition, $m_{\max}^{A_iA_j}$ is an increasing function of α_i for $\alpha_j \in]0, 1[$.

- Assuming $\alpha_i + \alpha_j > 1$ ($\epsilon_{A_iA_j} < 0$), $m_{\max}^{A_iA_j}$ is defined if $\text{Max}[1 - \alpha_i, \alpha_i] < \alpha_j < 1$. The maximum of $m_{\max}^{A_iA_j}$ is therefore not accessible and since $m_{\max}^{A_iA_j}$ is a decreasing function of α_j, its maximum is realized when $\alpha_j = \text{Max}[1 - \alpha_i, \alpha_i]$.
  - if $\alpha_i < .5$, then $\alpha_j \leq 1 - \alpha_i$. This leads to $m_{\max}^{A_iA_j} \leq \alpha_i < \frac{\Lambda_{\max}^{A_iA_j}}{2}$.
  - if $\alpha_i > .5$, then $\alpha_j \leq \alpha_i$. In particular, $\alpha_j = \alpha_i$ leads to $m_{\max}^{A_iA_j} = \alpha_i$ and $\epsilon_{A_iA_j} \to 1 - 2\alpha_i$. The genetic barrier formed with epistasis is stronger than the one formed without epistasis. In particular, if $\alpha_i = 1$, $\alpha_j = 1$ and $\epsilon_{A_iA_j} \to -1$, the genetic barrier is equal to the maximum amount of local adaptation, despite the two loci being in loose linkage.
- Assuming $\alpha_i + \alpha_j < 1$ ($\epsilon_{A_iA_j} > 0$), $m_{\max}^{A_iA_j}$ is defined if $\text{Max}[\alpha_i, \frac{1-\alpha_i}{2}] < \alpha_j \leq 1 - \alpha_i$.

– if $\alpha_i < .5$, then $.5 \in [\text{Max}[\alpha_i, \frac{1-\alpha_i}{2}], 1-\alpha_i]$. Therefore, the maximum of $m_{\text{max}}^{A_iA_j}$ is reached for $\alpha_j = .5$. Because $m_{\text{max}}^{A_iA_j}$ is an increasing function of α_i, the maximum is reached for $\alpha_i = .5$ and therefore the optimal situation corresponds to $\epsilon_{A_iA_j} = 0$.

– if $\alpha_i > .5$, then the maximum of $m_{\text{max}}^{A_iA_j}$ is reached for $\alpha_j = 1-\alpha_i$, leading to $\epsilon_{A_iA_j} = 0$.

Therefore, the optimal way to distribute $\Lambda_{\text{max}}^{A_iA_j}$ between α_i, α_j and $\epsilon_{A_iA_j}$ is the following: $\alpha_i = 1, \alpha_j = 1$ and $\epsilon_{A_iA_j} \to -1$. Note that if $\epsilon_{A_iA_j} = -1$, then the dynamical system does not admit a single point equilibrium, but an isocline $p_{A_j} = \frac{-\alpha_i+m+\alpha_i p_{A_i}}{\alpha_i(p_{A_i}-1)}$. All trajectories starting inside the simplex converge to this line.

S 1.2.4 Epistasis between two continental adaptations

1st option	a_iB_j	A_iB_j	a_ib_j	A_ib_j
	0	$\alpha_i + \epsilon_{A_iB_j}$	$-\beta_j$	$\alpha_i - \beta_j$
2nd option	B_kB_l	b_kB_l	B_kb_l	b_kb_l
	0	$-\beta_k - \epsilon_{B_kB_l}$	$-\beta_l - \epsilon_{B_kB_l}$	$-\beta_k - \beta_l - \epsilon_{B_kB_l}$

Table S2: **Fitness table and equivalence of a continental adaptation B_j interacting with an island adaptation, A_i, or two continental adaptations B_k and B_l interacting with each other.** Here, the continental haplotype has a fitness of zero on the island. Then, the two parametrizations are equivalent with the following change $\alpha_i = -\beta_k, \beta_j = \beta_l + \epsilon_{B_kB_l}$ and $\epsilon_{A_iB_j} = -\epsilon_{B_kB_l}$.

This case is equivalent to the case presented above, with the substitutions presented in Table S2. Therefore the optimal genetic barrier for a given $\Lambda_{\text{max}}^{b_kb_l}$ is given by $\beta_k = 0$, $\beta_l = 0$ and $\epsilon_{B_kB_l} \to -\Lambda_{\text{max}}^{b_kb_l}$. In this scenario, the role of the genetic barrier is to prevent the fixation of alleles B_k and B_l. With a limited amount of local adaptation available, the most effective approach to prevent this is to have strong selective pressure against B_k and B_l when they are close to fixation.

The definition of $\Lambda_{\text{max}}^{b_kb_l}$ is more cumbersome than in the previous scenarios: $\Lambda_{\text{max}}^{b_kb_l} = \text{Max}[-\beta_k, -\beta_l, -(\beta_k+\epsilon_{B_kB_l}), -(\beta_l+\epsilon_{B_kB_l}), -(\beta_k+\beta_l+\epsilon_{B_kB_l})]$. $\beta_l < 0$ and $\beta_k < 0$ are necessary conditions for the existence of the two-locus genetic barrier, leading to $\Lambda_{\text{max}}^{b_kb_l} = \text{Max}[-\beta_k, -\beta_l, -(\beta_k + \beta_l + \epsilon_{B_kB_l})]$.

If $\Lambda_{\text{max}}^{b_kb_l}$ is given by $-\beta_k$, i.e. $\beta_k < \beta_l$ and $\beta_l + \epsilon_{B_kB_l} > 0$, then (using Mathematica), it is easy to show that $m_{\text{max}}^{B_kB_l} \leq \frac{-\beta_k}{4} = \frac{\Lambda_{\text{max}}^{b_kb_l}}{4}$. Per symmetry, if $\Lambda_{\text{max}}^{b_kb_l}$ is given by $-\beta_k$, we obtain the same result.

If $\Lambda_{\text{max}}^{b_kb_l}$ is given by $-(\beta_k + \beta_l + \epsilon_{B_kB_l})$, then this case is identical to the 2 island adaptations case, the genetic barrier is the largest when $\beta_k \to 0$, $\beta_l \to 0$ and $\epsilon_{B_kB_l} = -\Lambda_{\text{max}}^{b_kb_l}$.

The best parameter combination to optimize the genetic barrier with a fixed $\Lambda_{\max}$ is $\beta_k = 0$, $\beta_l = 0$ and $\epsilon_{B_k B_l} \to -\Lambda_{\max}^{b_k b_l}$. In that case, $m_{\max}^{b_k b_l} \to \Lambda_{\max}^{b_k b_l}$.

S 1.3 3-locus model

S 1.3.1 One island mutation and two continental ones.

We now focus on the three-locus model. In the main manuscript, we demonstrated that $\Lambda_{\max} > m_{\max}^{AbC}$, with allele C appearing on the island, assuming that there were only two epistatic interactions between the derived alleles. In the following paragraph, we will focus on the case of allele C appearing on the continent.

- Negative pairwise epistasis between A and B and between A and C cannot generate a strong genetic barrier. Indeed, in the absence of allele C, we have $m_{\max}^{Ab|c} < \Lambda_{\max}^{Ab|c}$. Once allele C is introduced on the continent, it interacts negatively with allele A. The marginal fitness of allele A is therefore reduced. Allele A will be lost on the island at a lower migration rate, leading to $m_{\max}^{Abc} < m_{\max}^{Ab|c}$. Since the two-locus barrier is a subset of the three-locus barrier, we have $\Lambda_{\max}^{Ab|c} \leq \Lambda_{\max}^{Abc}$. Therefore we obtain $m_{\max}^{Abc} < \Lambda_{\max}^{Abc}$.

- Similarly, positive pairwise epistasis between A and B and between A and C cannot generate a strong genetic barrier. Indeed, in the absence of allele A, we have $m_{\max}^{bc|a} = \min(-\beta, -\gamma)$ while $\Lambda_{\max}^{bc|a} = -\beta - \gamma$. Since both β and γ are negative (otherwise there is no genetic barrier), we have $m_{\max}^{bc|a} < \Lambda_{\max}^{bc|a}$. Once allele A is introduced on the island, it increases the marginal fitness of allele B and C. As a consequence, allele B and C will fix on the island at a lower migration rate. We therefore obtain $m_{\max}^{Abc} < m_{\max}^{bc|a}$. Since the two-locus barrier is a subset of the three-locus barrier, we have $\Lambda_{\max}^{bc|a} \leq \Lambda_{\max}^{Abc}$. Therefore we obtain $m_{\max}^{Abc} < \Lambda_{\max}^{Abc}$.

- We now assume that one interaction is positive and the other negative. We assume that allele A interacts negatively with B and positively with C. In the absence of allele B, we know that for two loci with positive epistasis $m_{\max}^{Ac|b} \leq \Lambda_{\max}^{Ac|b}$. If we now assume that allele B exists on the island, then the marginal fitness of allele A is reduced due its association with allele B. We therefore obtain $m_{\max}^{Abc} \leq m_{\max}^{Ac|b}$. Since the two-locus barrier is a subset of the three-locus barrier, we have $\Lambda_{\max}^{Ac|b} \leq \Lambda_{\max}^{Abc}$. Therefore we obtain $m_{\max}^{Abc} < \Lambda_{\max}^{Abc}$.

- Finally, we assume that alleles A and B interact negatively and alleles B and C positively. In the absence of allele B, we know that for two loci without epistatic interaction, $m_{\max}^{Ac|b} \leq$

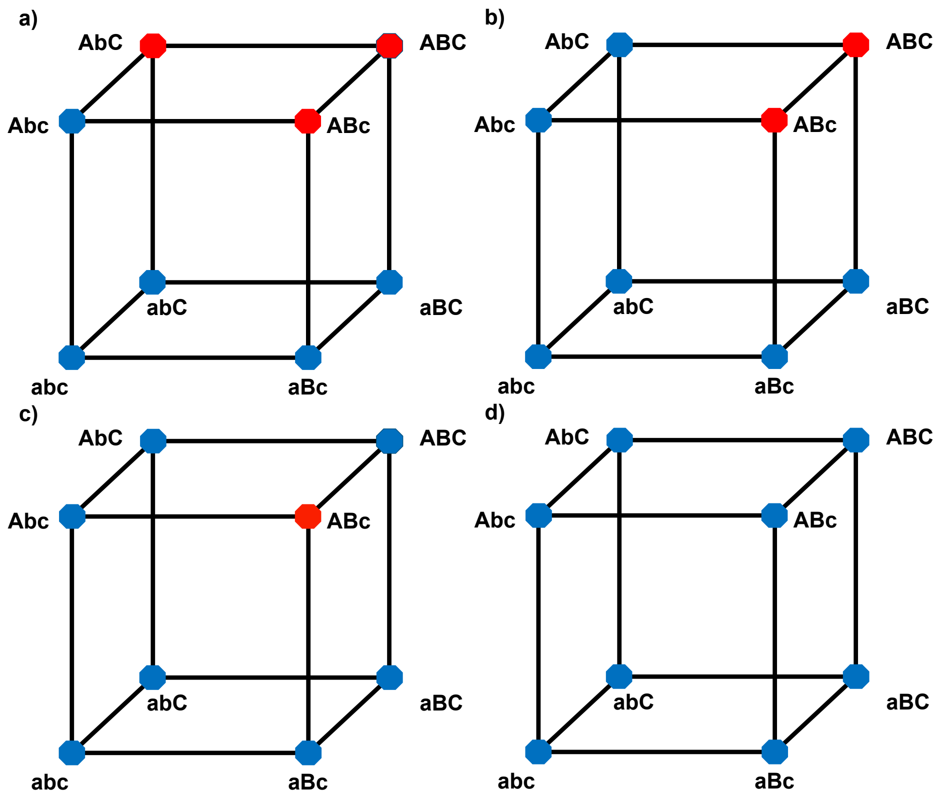

Figure S4: **Fitness graphs for the different scenarios with only two pairwise epistatic interactions** a) Allele C appears on the continent and there is negative epistasis between A and B and A and C. b) Alleles A and B interact negatively. There is positive epistasis between allele A and C or B and C but it is weak with regards to ϵ_{AB}. b) Alleles A and B interact negatively. There is positive epistasis between allele A and C or B and C and its strength is equivalent to ϵ_{AB}. d) The two epistatic interactions are positive (if C appears on the island, $\epsilon_{AB} > 0$ and $\epsilon_{bc} > 0$; if C appears on the continent, $\epsilon_{AB} > 0$ and $\epsilon_{AC} > 0$.

$\Lambda_{\max}^{Ac|b}$. If we now assume that allele B exists on the island, then the marginal fitness of allele A is reduced due its association with allele B. We therefore obtain $m_{\max}^{Abc} \leq m_{\max}^{Ac|b}$. Since the two-locus barrier is a subset of the three-locus barrier, we have $\Lambda_{\max}^{Ac|b} \leq \Lambda_{\max}^{Abc}$. Therefore we obtain $m_{\max}^{Abc} < \Lambda_{\max}^{Abc}$.

S 1.3.2 A genetic barrier can reach $\Lambda_{\max}$ even when all three loci are in loose linkage

We assume that there are three island adaptations A_1, A_1 and A_3. The direct effect of all adaptations is α. Pairwise epistasis between derived alleles is negative and given by ϵ. Three-locus positive epistasis exists between the three derived alleles and is given by $-\epsilon$. The whole fitness table is given in Table S3.

Using this model, the definition of the maximum amount of adaptation is: $\Lambda_{\max}^{A_1A_2A_3} = 3\alpha - 2\epsilon$. Unfortunately, this system has very complex equilibrium solutions so we could only calculate the strength of the genetic barrier numerically. Fig. S5 illustrates the strength of the resulting

Haplotype	$a_1a_2a_3$	$A_1a_2a_3$	$a_1A_2a_3$	$a_1a_2A_3$	$A_1A_2a_3$	$A_1a_2A_3$	$a_1A_2A_3$	$A_1A_2A_3$
Fitness	0	α	α	α	$2\alpha-\epsilon$	$2\alpha-\epsilon$	$2\alpha-\epsilon$	$3\alpha-2\epsilon$

Table S3: **Fitness table for the scenario of three island adaptations with $m_{\mathbf{max}} = \Lambda_{\mathbf{max}}$**

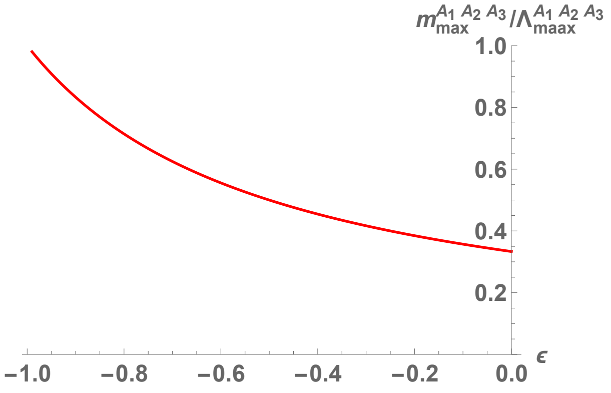

Figure S5: **Genetic barrier $m_{\mathbf{max}}^{A_1A_2A_3}$ as a function of epistasis.** The X axis corresponds to the strength of pairwise epistasis between the different island adaptations and the Y axis to the ratio of the strength of the genetic barrier to the amount of local adaptation.

genetic barrier relative to the amount of local adaptation. First, the genetic barrier is always smaller than the amount of local adaptation; however $m_{\text{max}}^{A_1A_2A_3} \underset{\epsilon\to-\alpha}{\to} \Lambda_{\text{max}}^{A_1A_2A_3}$. The genetic barrier is always the same and given by $m_{\text{max}}^{A_1A_2A_3} = \alpha$, despite the increasing negative epistasis. Epistasis does not affect the strength of the genetic barrier but the frequencies of the A_i alleles as illustrated in Fig. S6. Indeed epistasis plays a role only when the derived alleles are at high frequencies and therefore it has no effect on the genetic barrier, since the barrier is defined when those alleles are rare. The trend in Fig. S5 is due to the reduction of $\Lambda_{\text{max}}^{A_1A_2A_3}$ as epistasis increases and does not reflect a change in the strength of the genetic barrier. If $\epsilon = -\alpha$, then the solution of the system is a stable plane (1 negative eigenvalue and 2 eigenvalues equal to 0 for $m \leq \Lambda_{\text{max}}^{A_1A_2A_3}$).

Because the three-locus system is fully parameterized with 7 parameters, we obtain the equivalent result for different evolutionary histories: 2 island adaptations and a continental one, 2 continental adaptations and 1 island one, or three continental adaptations, see Table (S4). The first line for each model corresponds to the full parameterization of each model. As explained above, for the three islands adaptations, for simplicity we reduce the model to two parameters, selection (α) and epistasis (ϵ), making the model completely symmetric. Then we report the equivalent of this model for all other linkage architectures (2nd line for each model). Lastly, we focus on the condition to observe $m_{\text{max}}^{A_1A_2A_3} \to \Lambda_{\text{max}}^{A_1A_2A_3}$. One can notice that, if we reconstruct the fitness of each haplotype, we observe the following pattern: all haplotypes except the continental one have the exact same fitness, and the difference between the continental one

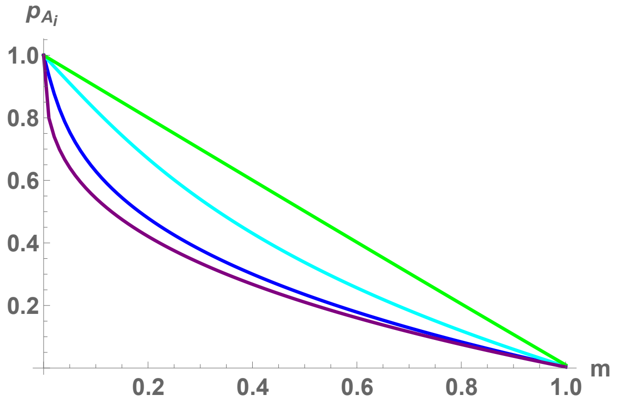

Figure S6: **Frequency of allele A_i as a function of migration.** The X-axis corresponds to the migration rate and the Y-axis to the frequency of alleles A_i. Due to symmetry, all alleles have the same frequencies at equilibrium. We assume $\alpha_i = 1$. The different colors correspond to different values of ϵ, the pairwise epistasis between island adaptations: green for -0.01, cyan -0.45, blue -0.85 and purple -0.99.

and all the others is $\Lambda_{\max}^{A_1A_2A_3}$.

3 Isl:	α_1	α_2	α_3	$\epsilon_{A_1A_2}$	$\epsilon_{A_1A_3}$	$\epsilon_{A_2A_3}$	$\epsilon_{A_1A_2A_3}$
2 param.	α	α	α	ϵ	ϵ	ϵ	$-\epsilon$
limit $\epsilon \to -\alpha$	α	α	α	$-\alpha$	$-\alpha$	$-\alpha$	α
3 Cont:	β_1	β_2	β_3	$\epsilon_{B_1B_2}$	$\epsilon_{B_1B_3}$	$\epsilon_{B_2B_3}$	$\epsilon_{B_1B_2B_3}$
2 param.	$-\alpha-\epsilon$	$-\alpha-\epsilon$	$-\alpha-\epsilon$	0	0	0	ϵ
limit $\epsilon \to -\alpha$	0	0	0	0	0	0	$-\alpha$
2 Isl and 1 Cont:	α_1	α_2	β	$\epsilon_{A_1A_2}$	ϵ_{A_1B}	ϵ_{A_2B}	$\epsilon_{A_1A_2B}$
2 param.	$\alpha+\epsilon$	$\alpha+\epsilon$	$-\alpha$	0	$-\epsilon$	$-\epsilon$	ϵ
limit $\epsilon \to -\alpha$	0	0	$-\alpha$	0	α	α	$-\alpha$
1 Isl and 2 Cont:	α	β_1	β_2	ϵ_{AB_1}	ϵ_{AB_2}	$\epsilon_{B_1B_2}$	$\epsilon_{AB_1B_2}$
2 param.	$\alpha+\epsilon$	$-\alpha-\epsilon$	$-\alpha-\epsilon$	0	0	ϵ	$-\epsilon$
limit $\epsilon \to -\alpha$	0	0	0	0	0	$-\alpha$	α

Table S4: **Equivalence between the different evolutionary histories, for the specific scenario $m_{\max} \to \Lambda_{\max}$**

S 2 Part II: the multi locus model

S 2.1 Model

In the main manuscript, we present the model for three loci, with epistasis existing between all derived alleles. Below, we present a general formulation for an arbitrary number of loci.

We consider diallelic loci classified into two classes of derived mutations, depending on their origin:

- Type-A mutations are locally adaptive on the island, but deleterious on the continent. Specifically, mutant allele A_i has a selective advantage α_i over the ancestral a_i allele on the island, with $i \in \{1, n_A\}$ and n_A the number of loci of type A. Type-A mutations always appear on the island and are absent from the continent.

- Type-B mutations are beneficial on the continent, where they appear and fix. On the island, mutant allele B_i has a selection coefficient β_i compared to the ancestral b_i allele on the island, with $i \in \{1, n_B\}$.

Epistasis can occur between any combination of derived alleles and is denoted by ϵ, with the involved alleles indicated as subscript. For example, $\epsilon_{A_iA_jB_k}$ denotes 3-way epistasis between two island-derived alleles A_i and A_j and one continental-derived allele B_k. As an example, the fitness table for a pair of A_i and B_j loci is given in Table S5.

Haplotype:	$a_ia_jb_k$	$A_ia_jb_k$	$a_iA_jb_k$	$a_ia_jB_k$	$A_iA_jb_k$	$A_ia_jB_k$	$a_iA_jB_k$	$A_iA_jB_k$
Direct effects	0	α_i	α_k	β_j	$\alpha_i + \alpha_j$	$\alpha_i + \beta_k$	$\alpha_j + \beta_k$	$\alpha_i + \alpha_j + \beta_k$
Pairwise epistasis					$+\epsilon_{A_iA_j}$	$+\epsilon_{A_iB_k}$	$+\epsilon_{A_jB_k}$	$+\epsilon_{A_iA_j} + \epsilon_{A_iB_k} + \epsilon_{A_jB_k}$
3rd order epistasis								$+\epsilon_{A_iA_jB_k}$

Table S5: **Fitness table for three loci A_i, A_j, and B_k, with the fitness effect decomposed according to the order of interaction.**

The fitness of an arbitrary haplotype $\omega(..A_i..a_j..b_k...B_l..)$ is given therefore by equation (S10).

$$
\begin{aligned}
\omega(..A_i..a_j..b_k...B_l..) &= \sum_i \alpha_i + \sum_l \beta_l \\
&+ \sum_{\substack{i,m \\ m\neq i}} \epsilon_{A_iA_m} + \sum_{i,l} \epsilon_{A_iB_l} + \sum_{\substack{l,m \\ m\neq l}} \epsilon_{B_lB_m} \\
&+ \sum_{\substack{i,m,n \\ m\neq i;n\neq i;n\neq m}} \epsilon_{A_iA_mA_n} + \sum_{\substack{i,l,m \\ m\neq i}} \epsilon_{A_iA_mB_l} + \sum_{\substack{i,l,m \\ m\neq l}} \epsilon_{A_iB_lB_m} + \sum_{\substack{l,m,n \\ m\neq l;n\neq l;n\neq m}} \epsilon_{B_lB_mB_n} \\
&+ ... \\
&+ \epsilon_{...A_i..B_l...}
\end{aligned}
\tag{S10}
$$

Due to the complexity of equation (S10), it is not possible to explicitly integrate this expression into a general formula beyond equation (1). However, if we assume that epistasis is only pairwise, then we can derive a more detailed expression for the whole system when all loci are in loose linkage:

$$
\begin{cases}
\dot{p_{A_i}} &= p_{A_i}((1-p_{A_i})(\alpha_i + \sum_{k=1,k\neq i}^{n_A} p_{A_k}\epsilon_{A_iA_k} + \sum_{k=1}^{n_B} p_{B_k}\epsilon_{A_iB_k}) - m) \\
\dot{p_{B_j}} &= p_{B_j}((1-p_{B_j})(\beta_j + \sum_{k=1,k\neq j}^{n_B} p_{B_k}\epsilon_{B_jB_k} + \sum_{k=1}^{n_A} p_{A_k}\epsilon_{A_kB_j}) - m) + m
\end{cases}
\tag{S11}
$$

S 2.2 Tight linkage case

As mentioned in the main manuscript, when all loci are in tight linkage, the system is equivalent to a single-locus model with $2^{n_A+n_B}$ alleles.

If all B-type mutations are deleterious on the island and only negative epistasis between continental and island adaptations exists ($\epsilon_{A_ib_j} \leq 0$, $\epsilon_{A_iAkb_j} \leq 0$, $\epsilon_{A_ib_jb_l} \leq 0...$), then the fittest haplotype on the island is $...A_i..b_j...$. The genetic barrier is always equal to the maximum amount of local adaptation, $m_{\max}^{...A_i..b_j..} = \Lambda_{\max}^{...A_i..b_j..}$.

Conversely, if we assume that there is at least one B_k mutation that is advantageous on the island compared to b_k and only negative epistasis between continental and island adaptations exists, the genetic barrier will always be smaller than the amount of local adaptation $m_{\max}^{...A_i...b_j...b_k...} < \Lambda_{\max}^{...A_i...b_j...b_k...}$. In that case, all $B_k, \beta_k > 0$ mutations must generate some negative epistasis with at least one A_i mutation, otherwise the genetic barrier $...A_i...b_j...b_k...$ does not exist. In this situation, stronger negative epistasis between B_k and A_i will never weaken the genetic barrier, but $m_{\max}^{...A_i..b_j..b_k..} < \Lambda_{\max}^{...A_i..d_j..b_k..}$. Indeed, in the absence of B_k mutations, we recover the results above. Adding B_k mutations reduces the fitness difference between the continental and the best island haplotypes; however $\Lambda_{\max}^{...A_i..d_j..b_k..}$ remains unchanged as it includes any subset of the genetic barrier, including the one without B_k mutations.

We now assume that we have $A_i, i \in I = \{1, 2, .., n_I\}$ island adaptations and $B_j, j \in J = \{1, 2, .., n_J\}$ continental adaptations. Continental adaptations can be advantageous or not on the island. Epistasis can only exist between any pair of derived alleles. If all loci are in tight linkage, this means that haplotype $..A_i..b_j.., i \in I, j \in J$ is the best haplotype on the island.

We define as $V \subset I$ a subset of island adaptations and as $O \subset J$ a subset of continental adaptations. Haplotype $..A_i..b_j..$ is the best haplotype, if for any subset V and O, the fitness of $..A_i..b_j..$ is higher than the fitness of $..A_i..a_v..B_o..b_j..$, leading to equation (S12).

$$\forall V \subset I, \forall O \subset J, -\sum_v^{v\in V} \alpha_v + \sum_o^{o\in O} \beta_o - \sum_v^{v\in V}(\sum_{k>v}^{k\in V} \epsilon_{A_v A_k} + \sum_k^{k\notin V} \epsilon_{A_v A_k}) + \sum_o^{o\in O} \sum_{k>o}^{k\in O} \epsilon_{b_o b_k} + \sum_k^{k\notin V} \sum_o^{o\in O} \epsilon_{a_k b_o} < 0 \tag{S12}$$

$$\forall O \subset J, \sum_o^{o\in O} \beta_o + \sum_k^{k\in I} \sum_o^{o\in O} \epsilon_{a_k b_o} < 0 \tag{S13}$$

In particular, if epistasis only happens between A_i and B_j loci and assuming $V = \varnothing$, equation (S12) reduces to equation (S13). If all B_j adaptations are deleterious and epistasis is only negative, it is obvious that equation (S13) is true.

S 2.3 Island adaptations in the absence of positive epistasis

We now consider that all loci are in loose linkage.

Any island adaptation A_i that is not involved in positive interactions will be swamped by gene flow if $m > \alpha_i$ [Blanckaert and Hermisson, 2018, Appendix C, eq. C15]. Indeed, using (S11) as a starting point, it is clear that if $m > \alpha$, the only solution to this equation is $p_{A_i} = 0$ since the maximum of $(1-p_{A_i})(\alpha_i + \sum_{k=1,k\neq i}^{n_A} p_{A_k}\epsilon_{A_i A_k} + \sum_{k=1}^{n_B} p_{B_k}\epsilon_{A_i B_k})$ is obtained for $p_A = 0$ and is less or equal α_i (and therefore $\dot{p_{A_i}} < 0$ for any value of p_{A_i} in $]0, 1]$) since all epistatic interactions are negative. Therefore, we have $m_{\max}^{...A_i..b_j...} \leq \alpha_i$. Including higher order negative epistasis makes this maximum even smaller and therefore does not change the result ($\dot{p_{A_i}} < 0$). The maximum amount of local adaptation is equal to or larger than α_i (because the fitness advantage of a haplotype that differs by a single island adaptation from the ancestral haplotype is always included in the maximum local adaptation). Therefore we obtain $m_{\max}^{...A_i...b_j...} < \Lambda_{\max}^{...A_i..b_j...}$.

S 2.4 Continental adaptations in the absence of negative epistasis

We consider that all loci are in loose linkage. If allele B_k is advantageous on the island, it will fix on the island as long as a single copy of the allele B_k is introduced. Therefore, under these conditions, no genetic barrier can be built including locus B_k. Any continental adaptation B_j, deleterious on the island and not involved in negative interactions, will fix on the island for $m > -\beta_j$ Blanckaert and Hermisson [2018]. Indeed, using similarly equation (S11), we can rewrite this expression as $m*(1-p_{B_j})+\beta_j p_{B_j}(1-p_{B_j})+p_{B_j}(1-p_{B_j})Ep$, with Ep corresponding to the various epistatic terms ($Ep \geq 0$). If $m > -\beta_j$, then it is clear that the previous expression is positive for any value of p_{B_j} in $[0,1[$. Therefore allele B_j will always fix, regardless of its starting frequency. Since $\Lambda_{\max}^{...A_i..b_j...} \geq \mathrm{Max}[\alpha_i, -\beta_j]$, we have $m_{\max}^{...A_i..b_j...} \leq \Lambda_{\max}^{...A_i..b_j...}$ for any barrier with at least a single locus of this type.

S 2.5 Only island adaptations

Assume next that divergence is entirely due to type-A mutations that originate on the island, with arbitrary interactions among the derived alleles. In loose linkage, all haplotypes that can be constructed from ancestral and derived alleles at the barrier loci, $\{a_i, A_i\}$, still exist on the island in the final state of the barrier (all A_i mutations have appeared on the island). The maximum amount of local adaptation $\Lambda_{\max}$ is the fitness difference between the continental haplotype ($a_1...a_n$) and the fittest of these haplotypes. It follows that the *marginal* fitness of any derived allele A_i that is part of the fittest haplotype can be, in the best case scenario, equal to the maximum amount of local adaptation $\Lambda_{\max}$ and otherwise must be smaller than $\Lambda_{\max}$, independently of the population composition. Since an allele is swamped from the island when gene flow is larger than its marginal fitness, we conclude that $\Lambda_{\max}$ is once again an upper bound to the barrier strength.

S 2.6 Only continental adaptations

Let us now consider a barrier formed only by type-B mutations such that the island haplotype $b_1..b_{n_B}$ has the highest intrinsic fitness value (this implies that all B_i are deleterious on the island in the ancestral background, $\beta_j < 0$). If all loci are in tight linkage, we have $m_{\max}^{..b_i..} \leq \Lambda_{\max}^{..b_i..}$. Due to recombination, allele b_i will no longer be associated with the best haplotype 100% of the time and will be found in other haplotypes of lower fitness. Its marginal fitness is therefore

decreased, leading to the fixation of allele B_i at a lower migration rate. Therefore $\Lambda_{\max}$ is again an upper bound to the barrier strength.

S 2.7 Only one type of epistasis

Let us now consider a genetic barrier formed by an arbitrary number of A_i and B_j mutations, all in loose linkage, and let us assume there is at least one mutation of each type (the case with no A_i or B_j has been covered above, sections S 2.5 and S 2.6).

We now assume that all epistatic interactions are negative. In that case, there is at least one allele A_i, that is not generating any positive epistasis. Based on the results derived above (section S 2.3), we can conclude that $m_{\max}^{...A_i..b_j...} \leq \alpha_i$. The maximum amount of local adaptation is equal to or larger than α_i (because the fitness advantage of a haplotype that differs by a single island adaptation from the ancestral haplotype is always included in the definition of the maximum amount of local adaptation). Therefore, we obtain $m_{\max}^{...A_i...b_j...} < \Lambda_{\max}^{...A_i..b_j...}$.

We now assume that all epistatic interactions are positive. In that case, there is at least one allele B_j, that is deleterious on the island and not generating any negative epistasis. Based on the results derived above (section S 2.4), we can then conclude that $m_{\max}^{...A_i..b_j...} \leq -\beta_j$. However, as mentioned above, the maximum amount of local adaptation is equal to or larger than $-\beta_j$ (because the fitness advantage of a haplotype that differs by a single continental adaptation from the ancestral haplotype is also always included in the definition of the maximum amount of local adaptation). Therefore, we obtain $m_{\max}^{...A_i...b_j...} < \Lambda_{\max}^{...A_i..b_j...}$.

S 2.8 Negative epistasis between 2 sets

We now assume that there are n_A island adaptations with arbitrary interactions among them and n_B continental adaptations, with only negative epistasis between the type-A and type-B mutations. Based on the previous paragraph, in the absence of the continental adaptations, we have $m_{\max}^{...A_i...|..b_j...} \leq \Lambda_{\max}^{...A_i...|..b_j...}$. Considering now that continental adaptations are present, the marginal fitness of the island adaptations is reduced due to the negative epistasis generated with the immigrating B_j alleles. Therefore, the genetic barrier is reduced, $m_{\max}^{...A_i..b_j..} \leq m_{\max}^{...A_i...|..b_j...}$. However, since having all b_j fixed is a subset of the general model, we have $\Lambda_{\max}^{..A_i..|..b_j..} \leq \Lambda_{\max}^{..A_i..b_j..}$. We therefore demonstrate that $\Lambda_{\max}$ is once again an upper bound to the strength of the genetic barrier.

If there are two (or more) disjoint sets of barrier loci that are mutually unlinked and not connected by epistasis of any order, the maximum amount of local adaptation $\Lambda_{\max}$ of the union of the two sets is always given by the sum of the $\Lambda_{\max}$ of the two subsets. In addition, the genetic barrier, $m_{\max}$, is determined by the weaker of the two subset barriers as the two sets of loci do not interact with each other (no linkage disequilibrium or any form of indirect selection). Therefore, if we are interested in the conditions under which a strong barrier exists despite limited local adaptation, we can always assume that all derived alleles are connected either by linkage or by epistasis (i.e., there should always be an unbroken path of interactions between any two derived alleles).

S 2.9 Illustration for 6 loci

To illustrate the general rules described above, we simulated the case of a 6-locus genetic barrier. With 6 diallellic loci, there are a total of 64 possible haplotypes, leading to a system of 63 equations (based on equation (1) in the main text). Such a system is impossible to solve (even numerically using Mathematica). We therefore used a custom C++ program to find the equilibrium state of a given population starting from a secondary contact situation (i.e. haplotype $A_1A_2A_3b_1b_2b_3$ starts fixed on the island). We iterate the continuous time equations until the sum of all absolute frequeny changes (for all 64 haplotypes) between two time steps becomes less than 10^{-10}. We start at a low migration rate and increase until we either end up with an equilibrium at which all 6 loci are not polymorphic or if the program did not converge after a million time steps (convergence can become slow when the leading eigenvalues are close to zero, indicating that a change in stability is likely to happen for a value of m very close to the currently investigated one). We assumed that the different loci are found in the following order along the chromosome: A_1, B_1, B_2, A_2, A_3 and B_3. We then consider two epistatic scenarios: A_1 interacts with B_1, A_2 with B_2 and A_3 with B_3 (Figure S7 left panels) and A_1 interacts with B_3, A_2 with B_1 and A_3 with B_2 (Figure S7 right panels). We start the simulations with haplotype $A_1A_2A_3b_1b_2b_3$ fixed on the island, as it corresponds to a secondary scenario case, and the most likely starting point to find a potential internal stable equilibrium. The resulting genetic barriers are shown in Figure S7. We recover our theoretical result: the strength of the genetic barrier cannot exceed the maximum amount of local adaptation. The source code can be found at `https://gitlab.com/evoldyn/strong_ri`.

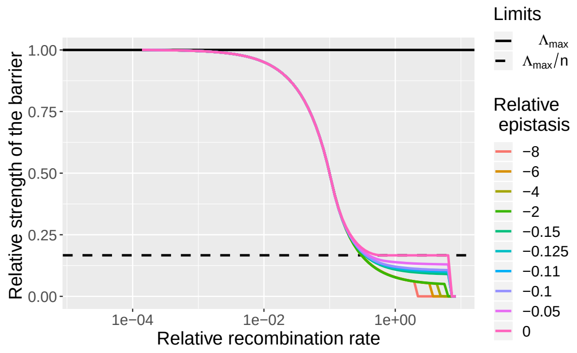

(a) $\alpha_1 = \alpha_2 = \alpha_3 = 0.01$, $\beta_1 = \beta_2 = \beta_3 = -0.01$

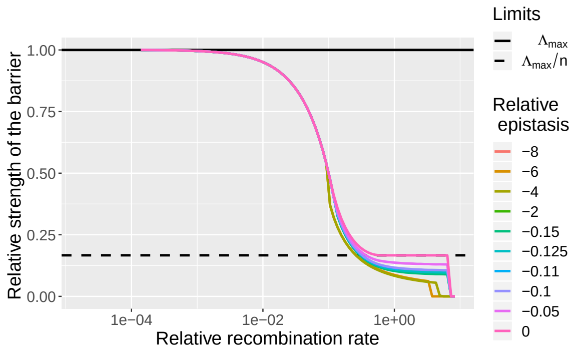

(b) $\alpha_1 = \alpha_2 = \alpha_3 = 0.01$, $\beta_1 = \beta_2 = \beta_3 = -0.01$

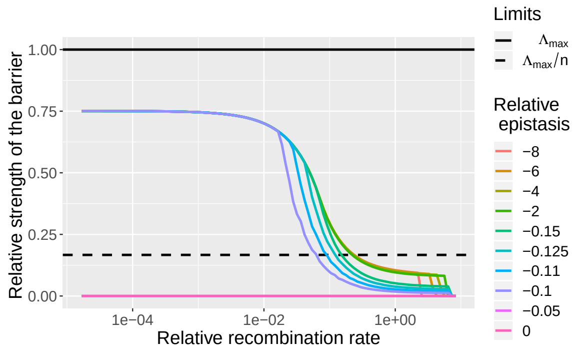

(c) $\alpha_1 = \alpha_2 = \alpha_3 = 0.02$ $\beta_1 = \beta_2 = \beta_3 = 0.05$

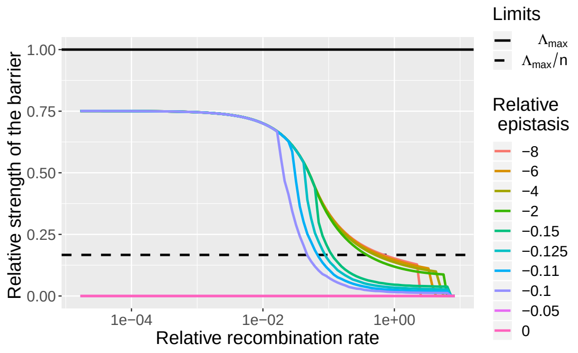

(d) $\alpha_1 = \alpha_2 = \alpha_3 = 0.02$ $\beta_1 = \beta_2 = \beta_3 = 0.05$

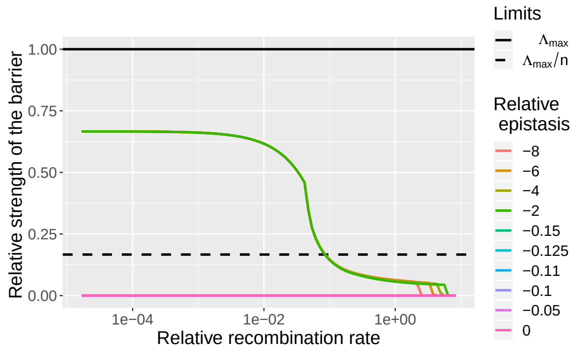

(e) $\alpha_1 = \alpha_2 = 0.01$, $\alpha_3 = 0.02$, $\beta_1 = 0.005$, $\beta_2 = -0.02$, $\beta_3 = 0.015$

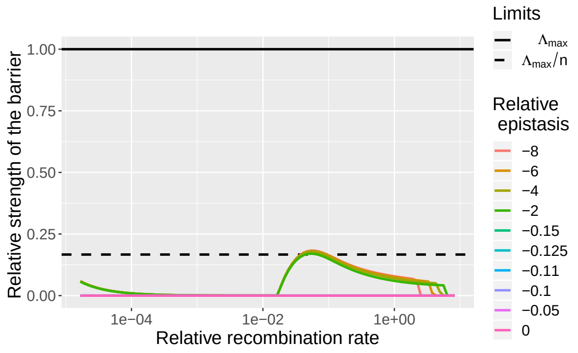

(f) $\alpha_1 = \alpha_2 = 0.01$, $\alpha_3 = 0.02$, $\beta_1 = 0.005$, $\beta_2 = -0.02$, $\beta_3 = 0.015$

Figure S7: **Strength of the genetic barrier relative to the amount of local adaptation** $\frac{m_{\mathbf{max}}^{A_1A_2A_3b_1b_2b_3}}{\Lambda_{\mathbf{max}}^{A_1A_2A_3b_1b_2b_3}}$. The black lines correspond to the limits derived in the main manuscript, with the solid line corresponding to all loci in tight linkage ($\Lambda_{\text{max}}^{A_1A_2A_3b_1b_2b_3}$) and the dashed line to all loci in loose linkage ($\Lambda_{\text{max}}^{A_1A_2A_3b_1b_2b_3}/6$). The colored lines correspond to simulations for 6 loci (100 points equally distributed on a log scale). For the left panels, pairwise negative epistasis happens between alleles of the same index (e.g., A_1 interacts with B_1, A_2 with B_2 and A_3 with B_3). For the right panels, pairwise negative epistasis happens between alleles A_1 and B_3, A_2 and B_1, and A_3 and B_2. All loci are equidistant and the recombination rate between adjacent loci scaled by Λ_{max} corresponds to the X axis. Each panel corresponds to a different sets of parameters chosen such as $\Lambda_{\text{max}}^{A_1A_2A_3b_1b_2b_3} = 0.06$.

S 2.10 The strength of genetic barrier can reach Λ_{max} with all loci in loose linkage

In this section, we show that a genetic barrier of independent loci (loci in loose linkage) can be as least as strong as a barrier of tightly linked loci for a given maximum amount of local adaptation Λ_{max}, given the right pattern of epistatic interaction among the derived alleles. This is a generalization of the example provided above for three loci (Section S 1.3.2). We have seen above that $m_{\mathrm{max}} = \Lambda_{\mathrm{max}}$ is an upper limit for the gene flow from the continent to the island to keep n tightly linked loci polymorphic. Below, we show that for any $m < \Lambda_{\mathrm{max}}$ also a barrier of n loci in loose linkage can be constructed that prevents swamping at these loci.

Barriers of this type can be constructed for any number of continental and island adaptations, but the proof is most straightforward if all adaptations (derived alleles) are on the island. Consider n loci in loose linkage, with ancestral alleles a_i fixed on the continent and derived alleles A_i segregating on the island with frequency p_i, $1 \leq i \leq n$. We assume the following fitness scheme: The fitness of the ancestral haplotype $(a_1, \ldots, a_n)$ on the island is normalized to 0, the fitness of the haplotype with all island alleles, $(A_1, \ldots, A_n)$, is Λ_{max} and the fitness of all other haplotypes is $\Lambda_{\mathrm{max}} - \epsilon$ for some small $\epsilon < \Lambda_{\mathrm{max}}$. Obviously, Λ_{max} is the maximum fitness deficit of continental invaders relative to optimal island individuals, conforming to our definition given in the model section of the main manuscript. We obtain a marginal fitness

$$w_i = \Lambda_{\mathrm{max}} - \epsilon + \epsilon \prod_{k \neq i} p_k$$

for all island alleles and a mean fitness in LE

$$\bar{w} = \Lambda_{\mathrm{max}} - \epsilon + \epsilon \prod_{k=1}^{n} p_k - (\Lambda_{\mathrm{max}} - \epsilon) \prod_{k=1}^{n} (1 - p_k) \,.$$

The dynamical equation for p_i reads

$$\dot{p}_i = -m p_i + (\Lambda_{\mathrm{max}} - \epsilon) p_i \prod_{k=1}^{n} (1 - p_k) + \epsilon p_i (1 - p_i) \prod_{k \neq i} p_k \,.$$

At an equilibrium, each p_i can either be zero or take some positive value. For the symmetric fully polymorphic equilibrium $p_i = p > 0$ we have

$$-m + (\Lambda_{\mathrm{max}} - \epsilon)(1 - p)^n + \epsilon (1 - p) p^{n-1} = 0 \,,$$

which has a unique solution $0 < p < 0.5$ for $m < \Lambda_{\max} - \epsilon$. Stability at this equilibrium follows from the Jacobian with off-diagonal elements

$$J_{i \neq j} = -(\Lambda_{\max} - \epsilon) p (1-p)^{n-1} + \epsilon p^{n-1} - \epsilon p^n$$

and diagonal elements

$$J_{ii} = -(\Lambda_{\max} - \epsilon) p (1-p)^{n-1} - \epsilon p^n \, .$$

The eigenvalues of this Jacobian are $J_{ii} - J_{i \neq j} = -\epsilon p^{n-1} < 0$ (as $(n-1)$-fold eigenvalue) and $n J_{i \neq j} < 0$ for small ϵ. For any $m < \Lambda_{\max}$, we thus find a sufficiently small $\epsilon < \Lambda_{\max} - m$ such that the fully polymorphic equilibrium is stable, which proves the claim. We note that for any fixed value of ϵ the monomorphic equilibrium $p_i = 0$ is stable for $m > \Lambda_{\max} - \epsilon$.

Via simple reparametrization of the fitness landscape, we obtain fully polymorphic equilibria for any combination of continental and island adaptations (and interactions only among derived alleles) up to the same limit $\Lambda_{\max}$. All these architectures require a combination of positive and negative epistasis of high order.

S 3 Part III: Cryptic epistasis

We now consider the scenario with cryptic epistasis with three loci, A, B and C, as introduced in the main text. In this part, we use the alternative notation introduced in the main manuscript: allele C generates a negative 3-locus epistasis ($\epsilon_{abC} < 0$) with the ancestral background ab with a selective advantage γ'.

S 3.1 Haploid populations

S 3.1.1 $m^{Ab|C}_{\mathbf{max}} > \Lambda^{AbC}_{\mathbf{max}}$ only if the internal equilibrium is locally stable.

Indeed, let us assume that such strong genetic barrier is also possible for a globally stable equilibrium (the genetic barrier corresponding to a globally stable equilibrium is denoted $m^{-,Ab|C}_{\max}$), i.e. $m^{-,Ab|C}_{\max} > \Lambda_{\max}$. Then at $m = m^{-,Ab|C}_{\max}$, the internal equilibrium is per definition no longer globally stable. Either the internal equilibrium leaves the state space through a boundary equilibrium, due to the loss of allele A or the fixation of allele B or it becomes locally stable, i.e. an internal equilibrium enters the state space through a boundary equilibrium. This implies that we have a single-locus polymorphism that can stably exist for a migration rate larger than the amount of local adaptation $\Lambda^{Ab|C}_{\max}$. Or the single-locus polymorphism is a subcase of the whole system, meaning that $\Lambda^{A}_{\max} \leq \Lambda^{Ab|C}_{\max}$. Or, as we have demonstrated above, $m^{A}_{\max} < \Lambda^{A}_{\max}$. Therefore, $m^{-,Ab|C}_{\max} > \Lambda_{\max}$ is impossible. The internal equilibrium can never be globally stable for $m > \Lambda^{Ab|C}_{\max}$. The strength of the genetic barrier can exceed the amount of local adaptation only if the internal equilibrium is locally stable. We can link this result to the mechanisms behind the genetic barrier: selection against hybrids in that case is the major force that shapes the genetic barrier. Or selection against hybrids is frequency-dependent, and therefore any equilibrium will be only locally stable, as the most common type represses the rare one.

S 3.1.2 Effect of the fixation of allele C on the genetic barrier

If A and B are in tight linkage, then the genetic barrier is given by:

$$\begin{aligned} &\text{Assuming} \quad \alpha > 0 \text{ and } \epsilon_{AB} < 0 \text{ and } \epsilon_{abC} < 0 \\ m^{Ab|C}_{\max} &= \alpha - \beta \quad \text{If } -\epsilon_{AB} > \beta \text{ and } \text{ and } \beta < \alpha + \gamma' \text{ and } \gamma' > 0 \end{aligned} \tag{S14}$$

While the conditions of existence of the genetic barrier are affected by allele C, the expression of $m^{Ab|C}_{\max}$ is identical to $m^{Ab|c}_{\max}$.

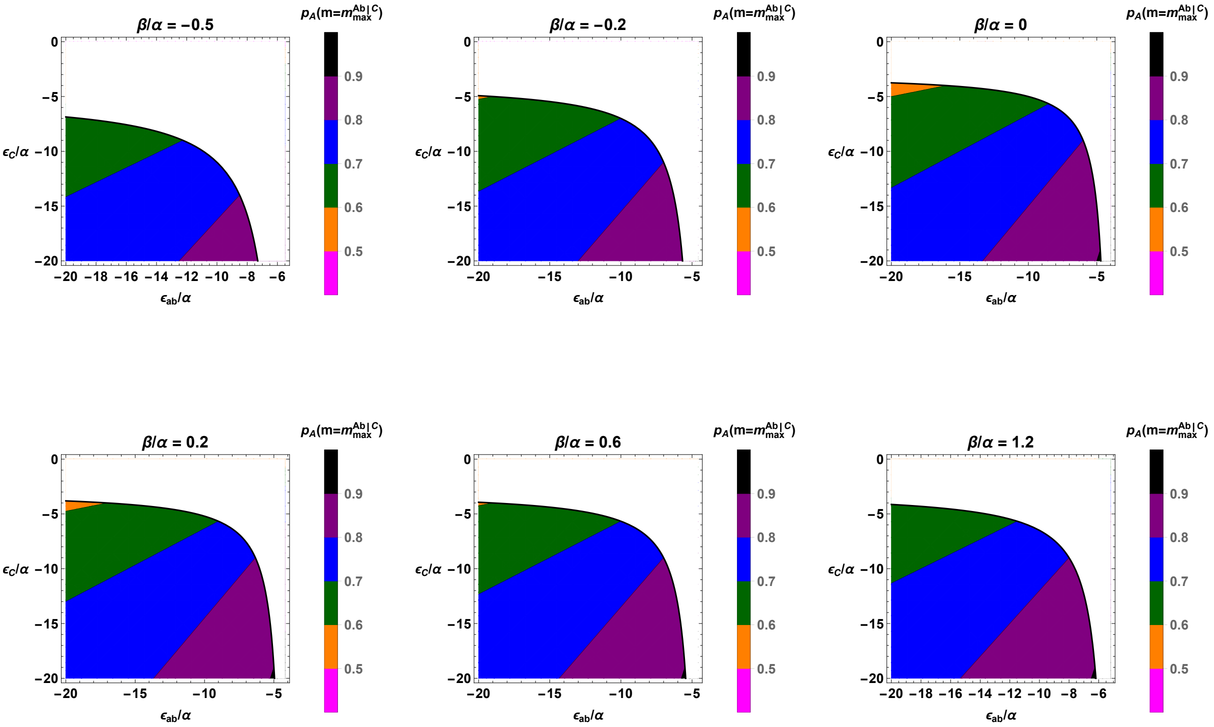

Figure S8: **Allele A remains the majority allele even at $m = m_{\mathbf{max}}^{Ab|C}$ in haploids, and in diploids with codominant epistasis.** We show the frequency of allele A when reaching $m_{\max}^{Ab|C}$ when the genetic barrier exceeds the maximum amount of local adaptation, assuming A and B are in loose linkage for different values of β.

If both loci are in loose linkage, then the expression for the strength genetic barrier is given in Bank et al. [2012] (equation (11)), after applying the substitutions given by:

$$\alpha_B = \alpha - \epsilon_{abC}, \beta_B = \beta - \epsilon_{abC} \text{ and } \gamma_B = -\epsilon_{AB} - \epsilon_{abC} \tag{S15}$$

S 3.1.3 The genetic barrier exceeds the amount of local adaptation

If both loci are in loose linkage, then the strength of the genetic barrier can exceed $\Lambda_{\max}^{2L}$ only if the genetic barrier corresponds to the two internal equilibria colliding with each other as demonstrated in section S 3.1.1; the expression is given by:

$$m_{\max}^{Ab|C} = \frac{(\alpha - \epsilon_{abC})(\epsilon_{AB} + \beta)}{4(\epsilon_{AB} + \epsilon_{abC})} \quad \text{if} \quad \epsilon_{AB} < -\text{Max}[(\alpha + \epsilon_{abC})/2, |\beta - \epsilon_{abC}| + \epsilon_{abC}] \tag{S16}$$

Using Mathematica, we calculate the necessary conditions to observe $m_{\max}^{Ab|C} > \Lambda_{\max}^{Ab|C}$ given in equation (5) in the main manuscript.

Figure S8 shows that allele A remains the majority allele in all cases when the genetic barrier is stronger than the maximum amount of local adaptation.

S 3.2 Diploid populations

S 3.2.1 Model

S 3.2.1.1 Dominance scheme For diploid populations, we first need to define the dominance scheme. Indeed, when compared to an haploid individual, a diploid model has an extra level of complexity: there are not only potential interactions between loci but also between the 2 alleles at the loci themselves. Following Bank et al. [2012] and as explained in the main manuscript, we assume that all direct effects are additive. Epistatic effects can have arbitrary levels of dominance. For the DMI between A and B, we use the following notation: η_{AB} for the double heterozygote, ϵ_{AB} if one locus is heterozygous and the other homozygous for the derived state and θ_{AB} if both loci are homozygous for the derived state Blanckaert and Hermisson [2018]. For the three locus epistatic interaction, we assume that there is potential epistasis as soon as an ancestral allele at locus A or B and a derived one at locus C are in contact, i.e. allele C is in contact with allele a or b. The fitness scheme is given in Table S6. The term ϵ_{abCijk} corresponds to this 3-locus epistasis component, with i, j and k counting the number of derived alleles at the loci, A, B, and C.

S 3.2.1.2 Conditions under which the system of equations is analytically solvable First, we assume that A and B are in loose linkage. We derive the conditions, given in equation (S17) under which we can analytically solve the full system. These conditions can be summarized as follows: if A (resp. B) is homozygous, then going from one derived allele B (resp. A) to none increases the amount of epistasis generated by a factor 2. In addition, the epistasis generated by the double heterozygote $AaBb$ has to be equal to the mean epistasis generated by the two single homozygotes (i.e. the epistasis generated by $AaBB$ and $Aabb$ as well as the epistasis generated by $aaBb$ and $AABb$). The last condition corresponds to the epistasis generated by the double homozygote $AABB$ corresponding to the sum of the transition from the double heterozygotes to the single heterozygote. Note that the classical 2 locus with a codominant DMI model, defined in Bank et al. [2012] fulfills these conditions.

	abc	Abc	aBc	ABc	abC	AbC	aBC	ABC
abc	0	α	β	$\alpha+\beta$ $+\eta_{AB}$	γ' $+\epsilon_{abC001}$	$\alpha+\gamma'$ $+\epsilon_{abC101}$	$\beta+\gamma'$ $+\epsilon_{abC011}$	$\alpha+\beta+\gamma'$ $+\eta_{AB}+\epsilon_{abC111}$
Abc		2α	$\alpha+\beta$ $+\eta_{AB}$	$2\alpha+\beta$ $+\epsilon_{AB}$	$\alpha+\gamma'$ $+\epsilon_{abC101}$	$2\alpha+\gamma'$ $+\epsilon_{abC201}$	$\alpha+\beta+\gamma'$ $+\eta_{AB}+\epsilon_{abC111}$	$2\alpha+\beta+\gamma'$ $+\epsilon_{AB}+\epsilon_{abC211}$
aBc			2β	$\alpha+2\beta$ $+\epsilon_{AB}$	$\beta+\gamma'$ $+\epsilon_{abC011}$	$\alpha+\beta+\gamma'$ $+\eta_{AB}+\epsilon_{abC111}$	$2\beta+\gamma'$ $+\epsilon_{abC021}$	$\alpha+2\beta+\gamma'$ $+\epsilon_{AB}+\epsilon_{abC121}$
ABc				$2\alpha+2\beta$ $+\theta_{AB}$	$\alpha+\beta+\gamma'$ $+\eta_{AB}+\epsilon_{abC111}$	$2\alpha+\beta+\gamma'$ $+\epsilon_{AB}+\epsilon_{abC211}$	$\alpha+2\beta+\gamma'$ $+\epsilon_{AB}+\epsilon_{abC121}$	$2\alpha+2\beta+\gamma'$ $+\theta_{AB}+\epsilon_{abC221}$
abC					$2\gamma'$ $+\epsilon_{abC002}$	$\alpha+2\gamma'$ $+\epsilon_{abC102}$	$\beta+2\gamma'$ $+\epsilon_{abC012}$	$\alpha+\beta+2\gamma'$ $+\eta_{AB}+\epsilon_{abC112}$
AbC						$2\alpha+2\gamma'$ $+\epsilon_{abC202}$	$\alpha+\beta+2\gamma'$ $+\eta_{AB}+\epsilon_{abC112}$	$2\alpha+\beta+2\gamma'$ $+\epsilon_{AB}+\epsilon_{abC212}$
aBC							$2\beta+2\gamma'$ $+\epsilon_{abC022}$	$\alpha+2\beta+2\gamma'$ $+\epsilon_{AB}+\epsilon_{abC122}$
ABC								$2\alpha+2\beta+2\gamma'$ $+\theta_{AB}$

Table S6: **Fitness table for the 3-locus epistasis model, assuming fixation of allele C at locus C.**

$$\begin{cases} \epsilon_{abC022} & = 2\epsilon_{abC122} + 2\epsilon_{AB} - \theta_{AB} \\ \epsilon_{abC202} & = 2\epsilon_{abC212} + 2\epsilon_{AB} - \theta_{AB} \\ \epsilon_{abC102} & = 2\epsilon_{C112} - \epsilon_{abC122} + 2\eta_{AB} - \epsilon_{AB} \\ \epsilon_{abC012} & = 2\epsilon_{abC112} - \epsilon_{abC212} + 2\eta_{AB} - \epsilon_{AB} \\ \epsilon_{abC002} & = 4\epsilon_{abC112} - 2(\epsilon_{abC122} + \epsilon_{abC212}) + \theta_{AB} - 4\epsilon_{AB} + 4\eta_{AB} \end{cases} \tag{S17}$$

In particular, assuming that the DMI between A and B is codominant (but this is not a necessary condition), this system reduces to the classical codominant 2-locus model, presented in Bank et al. [2012].

Next, we consider that both A and B are in tight linkage. We apply the same reasoning. We derive the conditions for this system to reduce to a two-locus model with loci in tight linkage. These conditions are given in equation (S18).

$$\begin{cases} \epsilon_{abC012} = & \frac{\epsilon_{abC002}+\epsilon_{abC022}}{2} \\ \epsilon_{abC122} = & \frac{\epsilon_{abC022}-\epsilon_{abC202}}{2} + \epsilon_{abC212} \\ \epsilon_{abC102} = & \frac{\epsilon_{abC002}+\epsilon_{abC202}}{2} \end{cases} \tag{S18}$$

S 3.2.1.3 Recessivity and codominance Whereas we define the model for arbitrary levels of dominance, we focus on two specific cases of dominance that are biologically relevant:

- A recessive case, where the 3-locus incompatibility is expressed if there are two copies of allele C and at least one of alleles a and b or two copies of alleles a and b and at least one of allele C , i.e. all $\epsilon_{abCijk} = 0$ but $\epsilon_{abC002} = 2\epsilon_{abC}, \epsilon_{abC102} = \epsilon_{abC012} = \epsilon_{abC001} = \epsilon_{abC}$. The interaction between A and B is given by $\eta_{AB} = 0$ and $\theta_{AB} = 2\epsilon_{AB}$. Both the tight linkage and loose linkage models reduce to the diploid recessive model presented in Bank et al. [2012] with the proper substitutions.

- A codominant case, which, under the assumption of loose linkage, is equivalent to the haploid case. The list of substitutions needed to obtain the codominant model is given in equation (S19). Both the tight linkage and loose linkage models reduce to the model presented in Bank et al. [2012] with the appropriate substitutions.

$$\begin{aligned}
&\epsilon_{abC001} \to \epsilon_{abC}, \epsilon_{abC002} \to 2\epsilon_{abC}, \epsilon_{abC011} \to \tfrac{\epsilon_{abC}}{2}, \epsilon_{abC012} \to \epsilon_{abC}, \epsilon_{abC021} \to 0, \epsilon_{abC022} \to 0\\
&\epsilon_{abC101} \to \tfrac{\epsilon_{abC}}{2}, \epsilon_{abC102} \to \epsilon_{abC}, \epsilon_{abC111} \to \tfrac{\epsilon_{abC}}{4}, \epsilon_{abC112} \to \tfrac{\epsilon_{abC}}{2}, \epsilon_{abC121} \to 0, \epsilon_{abC122} \to 0\\
&\epsilon_{abC201} \to 0, \epsilon_{abC202} \to 0, \epsilon_{abC211} \to 0, \epsilon_{abC212} \to 0, \eta_{AB} \to \tfrac{\epsilon_{AB}}{2}, \theta_{AB} \to 2\epsilon_{AB}
\end{aligned} \tag{S19}$$

S 3.2.1.4 Definition of the amount of local adaptation Similar to the haploid model, we need to define the amount of local adaptation for the diploid model. The amount of local adaptation is obtained by comparing the optimal genotype (which is homozygous since we do not have any overdominance or heterozygote advantage) to the continental genotype corrected by ploidy. We exclude the three incompatible homozygous genotypes: abC/abC, ABc/ABc and ABC/ABC. We obtain the following expression for the amount of local adaptation: $\Lambda_{\max}^{Ab|C} = \text{Max}(\alpha, \alpha - \beta, \alpha - \beta - \gamma', -\gamma')$. This is the exact same expression as in the haploid model,

$$(\beta < 0 \text{ and } \gamma' \leq 0) \text{ or } (\beta > 0 \text{ and } -\gamma' < \text{Min}[\alpha, \beta]). \tag{S20}$$

S 3.2.2 Tight linkage and codominant interactions

Equation (S21) details under which condition we can observe a genetic barrier, the strength of which is larger than the maximum amount of local adaptation, for a diploid codominant incompatibility with A and B in tight linkage.

$$
\beta < 0 \begin{cases} \text{If cond}_1 & \epsilon_{abC} < \frac{1}{7}(6\alpha + 2\beta + \epsilon_{AB}) \\ \text{If cond}_2 & \epsilon_{abC} < \frac{1}{6}\left(6\alpha + \sqrt{12\alpha^2 + 12\alpha(\epsilon_{\mathrm{AB}} - \beta) - 10\beta\epsilon_{\mathrm{AB}} + \epsilon_{\mathrm{AB}}^2 + \beta^2} + \epsilon_{\mathrm{AB}} + \beta\right) \\ \text{If cond}_3 & \epsilon_{abC} < \frac{2\beta(\alpha+\beta)+(\alpha+6\beta)\epsilon_{\mathrm{AB}}+3\epsilon_{\mathrm{AB}}^2}{\alpha+\epsilon_{\mathrm{AB}}} \\ \text{If cond}_4 & \epsilon_{abC} < 6\beta - \left(4\sqrt{2}(\alpha-\beta) + 6\alpha + \epsilon_{\mathrm{AB}}\right) \end{cases}
$$

$$
\beta > 0 \begin{cases} \text{If cond}_5 & \epsilon_{abC} < \frac{1}{6}\left(6\alpha + \sqrt{12\alpha^2 + 12\alpha\epsilon_{\mathrm{AB}} + (\epsilon_{\mathrm{AB}} + 2\beta)^2} + \epsilon_{\mathrm{AB}} + 2\beta\right) \\ \text{If cond}_6 & \epsilon_{abC} < 2\beta - \epsilon_{\mathrm{AB}}\left(\frac{2\alpha}{\alpha+\epsilon_{\mathrm{AB}}+\beta} - 3\right) \\ \text{If cond}_7 & \epsilon_{abC} < -4\sqrt{2}\sqrt{\alpha(\alpha+\beta)} - 6\alpha - \epsilon_{\mathrm{AB}} - 2\beta \\ \text{If cond}_8 & \epsilon_{abC} < \frac{1}{7}(6\alpha + \epsilon_{\mathrm{AB}} + 2\beta) \\ \text{If cond}_9 & \epsilon_{abC} < 2\alpha + 7\epsilon_{\mathrm{AB}} + 6\beta \end{cases}
$$

$$
\begin{aligned}
cond_1 = {} & \left(\tfrac{1}{23}\left(7\sqrt{2}(\epsilon_{\mathrm{AB}} + \beta) - 12\epsilon_{\mathrm{AB}} + 11\beta\right) = \alpha \| \alpha < \tfrac{1}{23}\left(7\sqrt{2}(\epsilon_{\mathrm{AB}} + \beta) - 12\epsilon_{\mathrm{AB}} + 11\beta\right)\right) \\
& \&12\alpha^2 + 12\alpha(\epsilon_{\mathrm{AB}} - \beta) - 10\beta\epsilon_{\mathrm{AB}} + \epsilon_{\mathrm{AB}}^2 + \beta^2 > 0 \\
& \&\tfrac{1}{7}(6\alpha + \epsilon_{\mathrm{AB}} + 2\beta) > \tfrac{1}{6}\left(6\alpha + \sqrt{12\alpha^2 + 12\alpha(\epsilon_{\mathrm{AB}} - \beta) - 10\beta\epsilon_{\mathrm{AB}} + \epsilon_{\mathrm{AB}}^2 + \beta^2} + \epsilon_{\mathrm{AB}} + \beta\right) \\
cond_2 = {} & \alpha < \tfrac{1}{23}\left(7\sqrt{2}(\epsilon_{\mathrm{AB}} + \beta) - 12\epsilon_{\mathrm{AB}} + 11\beta\right)\&12\alpha^2 + 12\alpha(\epsilon_{\mathrm{AB}} - \beta) - 10\beta\epsilon_{\mathrm{AB}} + \epsilon_{\mathrm{AB}}^2 + \beta^2 > 0 \\
cond_3 = {} & \alpha + \epsilon_{\mathrm{AB}} < 0\&\sqrt{2}(\epsilon_{\mathrm{AB}} + \beta) < \alpha + 2\epsilon_{\mathrm{AB}} + \beta \\
cond_4 = {} & \tfrac{1}{23}\left(7\sqrt{2}(\epsilon_{\mathrm{AB}} + \beta) - 12\epsilon_{\mathrm{AB}} + 11\beta\right) < \alpha\&\alpha + 2\epsilon_{\mathrm{AB}} + \beta \leq \sqrt{2}(\epsilon_{\mathrm{AB}} + \beta) \\
cond_5 = {} & \tfrac{1}{7}(6\alpha + \epsilon_{\mathrm{AB}} + 2\beta) < \tfrac{1}{6}\left(6\alpha + \sqrt{12\alpha^2 + 12\alpha\epsilon_{\mathrm{AB}} + (\epsilon_{\mathrm{AB}} + 2\beta)^2} + \epsilon_{\mathrm{AB}} + 2\beta\right) \\
& \&\tfrac{7\sqrt{\alpha(\alpha+\beta)}}{\sqrt{2}} + 6\alpha + \epsilon_{\mathrm{AB}} + 2\beta < 0 \\
cond_6 = {} & 2\alpha + \sqrt{8\beta\epsilon_{\mathrm{AB}} + 8\epsilon_{\mathrm{AB}}^2 + \beta^2} + 4\epsilon_{\mathrm{AB}} + 3\beta > 0\&\alpha + \epsilon_{\mathrm{AB}} + \beta < 0 \\
cond_7 = {} & \left(7\sqrt{2}\sqrt{\alpha(\alpha+\beta)} + 12\alpha + 2\epsilon_{\mathrm{AB}} + 4\beta > 0\&\sqrt{2}\sqrt{\alpha(\alpha+\beta)} + 2\alpha + 2(\epsilon_{\mathrm{AB}} + \beta) < 0\right. \\
& \left.\&2\alpha + 7\epsilon_{\mathrm{AB}} + 6\beta < -4\sqrt{2}\sqrt{\alpha(\alpha+\beta)} - 6\alpha - \epsilon_{\mathrm{AB}} - 2\beta\right) \| \left(\tfrac{7\sqrt{\alpha(\alpha+\beta)}}{\sqrt{2}} + 6\alpha + \epsilon_{\mathrm{AB}} + 2\beta > 0\right. \\
& \left.\&2\sqrt{2}\sqrt{\alpha(\alpha+\beta)} + 3\alpha + \epsilon_{\mathrm{AB}} + 2\beta < 0\&\epsilon_{\mathrm{AB}} + 2\beta < -4\sqrt{2}\sqrt{\alpha(\alpha+\beta)} - 6\alpha - \epsilon_{\mathrm{AB}} - 2\beta\right) \\
cond_8 = {} & \left(\tfrac{7\sqrt{\alpha(\alpha+\beta)}}{\sqrt{2}} + 6\alpha + \epsilon_{\mathrm{AB}} + 2\beta \leq 0\&2\alpha + 7\epsilon_{\mathrm{AB}} + 6\beta < \tfrac{1}{7}(6\alpha + \epsilon_{\mathrm{AB}} + 2\beta)\right) \\
& \| \left(\epsilon_{\mathrm{AB}} + 2\beta < \tfrac{1}{7}(6\alpha + \epsilon_{\mathrm{AB}} + 2\beta)\&\tfrac{7\sqrt{\alpha(\alpha+\beta)}}{\sqrt{2}} + 6\alpha + \epsilon_{\mathrm{AB}} + 2\beta \leq 0\right) \\
cond_9 = {} & 2\alpha + \sqrt{8\beta\epsilon_{\mathrm{AB}} + 8\epsilon_{\mathrm{AB}}^2 + \beta^2} + 4\epsilon_{\mathrm{AB}} + 3\beta \leq 0
\end{aligned}
\tag{S21}
$$

S 3.3 Fixation of allele C on the island, for the 3-locus epistasis model

For the scenario with cryptic epistasis, we always assume that C appears on the continent and fixes rapidly on the island. Here we derive and illustrate the condition for C to indeed fix

on the island. Fixation of allele C depends on the position of allele C in the genome. Here we will provide these conditions for all linkage architectures and dominance schemes.

Equation (S22) gives the minimal selective advantage $\gamma'_{\min}$ for an allele C to fix on the island in the codominant model, with all loci in loose linkage. It also corresponds to the haploid case; as the two models share the same dynamics.

$$\begin{aligned}\gamma'_{\min} &= -m - \epsilon_{abC}(1-p_A)(1-p_B)\\ \gamma'_{\min} &= \frac{\epsilon_{abC}(\alpha+\epsilon_{AB})(\beta+\epsilon_{AB})\left(\sqrt{\frac{(\alpha-\epsilon_{abC})(\beta+\epsilon_{AB})-4m(\epsilon_{AB}+\epsilon_{abC})}{(\alpha-\epsilon_{abC})(\beta+\epsilon_{AB})}}-1\right)}{2(\epsilon_{AB}+\epsilon_{abC})^2} - \frac{m\epsilon_{AB}}{\epsilon_{AB}+\epsilon_{abC}}\end{aligned} \tag{S22}$$

For the recessive case, with all loci in loose linkage, the fixation condition for allele C is given in equation (S23). However, we did not obtain an analytical expression for the equilibrium of the 2 locus system and therefore relied on numerical evaluation.

$$\gamma'_{\min} = -m + (p_A-1)(p_B-1)\epsilon_{abC}(3p_Ap_B - p_A - p_B - 1) \tag{S23}$$

If all loci are in tight linkage, we know that the genetic barrier can only be exceeded if the epistatic interactions are codominant. Equation (S24) gives the minimal value of γ' for fixation of allele C. There are 4 different conditions, depending on which background is the most favourable for the invasion of allele c: the first one corresponds to ab, the second to Ab, the third to aB and the last to AB.

$$\begin{aligned}\gamma'_{\min} = \;& \text{Max}\;\Big[\tfrac{1}{2}(-2\beta - 2m + 2p_A(-\alpha+\beta+(p_A-1)(\epsilon_{AB}+\epsilon_{abC})) + \epsilon_{abC}),\\ & \tfrac{1}{4}((p_A-1)(-4\alpha+4\beta+4p_A\epsilon_{AB}+4p_A\epsilon_{abC}-2\epsilon_{AB}-\epsilon_{abC})-4m),\\ & \tfrac{1}{4}p_A(-4\alpha+4\beta+4p_A\epsilon_{AB}+4p_A\epsilon_{abC}-2\epsilon_{AB}-3\epsilon_{abC})-m,\\ & \alpha - m - \alpha p_A + p_A(\beta+(p_A-1)(\epsilon_{AB}+\epsilon_{abC})) + \epsilon_{AB}\Big]\\ \text{With} & \\ p_A = \;& -\frac{-2\alpha+2\beta+\sqrt{(-2\alpha+2\beta+\epsilon_{AB}+\epsilon_{abC})^2+16m(\epsilon_{AB}+\epsilon_{abC})}-3(\epsilon_{AB}+\epsilon_{abC})}{4(\epsilon_{AB}+\epsilon_{abC})}\end{aligned} \tag{S24}$$

Equation (S25) gives the condition for invasion of allele c if locus C is in loose linkage and A and B are in tight linkage. Similar to the previous case where all loci where in tight linkage, if the epistatic interactions are recessive, the genetic barrier can never exceed Λ_{max} and therefore

this case is not investigated.

$$
\begin{aligned}
\gamma'_{\min} = \;& -m - \tfrac{\epsilon_{abC}}{2} p_A(1-p_A) \\
\gamma'_{\min} = \;& -\tfrac{\epsilon_{abC}(2\alpha-2\beta+\epsilon_{AB}-\epsilon_{abC})}{16(\epsilon_{AB}-\epsilon_{abC})^2}\left(2\alpha-2\beta-\sqrt{(2\alpha-2\beta-\epsilon_{AB}+\epsilon_{abC})^2+16m(\epsilon_{AB}-\epsilon_{abC})}-\epsilon_{AB}+\epsilon_{abC}\right) \\
& + \tfrac{8m(\epsilon_{abC}-2\epsilon_{AB})(\epsilon_{abC}-\epsilon_{AB})}{16(\epsilon_{AB}-\epsilon_{abC})^2}
\end{aligned} \tag{S25}
$$

Assuming B and C are in tight linkage and A is in loose linkage, equation (S26) gives the minimal value of γ' to ensure fixation of C on the island if the epistatic interactions are codominant. The first condition corresponds to allele c invading in the background of allele b and the second one to invasion of allele c in the background of B, if B is advantageous enough.

$$
\begin{aligned}
\text{If } 2\beta \leq \;& \epsilon_{abC}(1-p_A) - 2p_A\epsilon_{AB} \\
& \gamma'_{\min} = -m - p_B(\beta + p_A\epsilon_{AB}) - \tfrac{1}{2}(-1+p_A)(-2+3p_B)\epsilon_{abC} \\
\text{If } 2\beta > \;& \epsilon_{abC}(1-p_A) - 2p_A\epsilon_{AB} \\
& \gamma'_{\min} = -\tfrac{1}{2}(2m + (p_B-1)(2\beta + 2p_A\epsilon_{AB} - 3\epsilon_{abC}(1-p_A)))
\end{aligned} \tag{S26}
$$

With

$$
\begin{aligned}
p_A = \;& \frac{(\epsilon_{abC}-\alpha)(\beta-\epsilon_{AB}-2\epsilon_{abC})-\sqrt{(\alpha-\epsilon_{abC})(\beta+\epsilon_{AB})((\alpha-\epsilon_{abC})(\beta+\epsilon_{AB})-4m(\epsilon_{AB}+\epsilon_{abC}))}}{2(\alpha-\epsilon_{abC})(\epsilon_{AB}+\epsilon_{abC})} \\
p_B = \;& 1 - \frac{(\beta+\epsilon_{AB})(\alpha+2\epsilon_{AB}+\epsilon_{abC})+\sqrt{(\alpha-\epsilon_{abC})(\beta+\epsilon_{AB})((\alpha-\epsilon_{abC})(\beta+\epsilon_{AB})-4m(\epsilon_{AB}+\epsilon_{abC}))}}{2(\beta+\epsilon_{AB})(\epsilon_{AB}+\epsilon_{abC})}
\end{aligned}
$$

If the epistatic interactions are recessive, equation (S27) gives the minimal value of γ' to ensure fixation of allele C on the island. As mentioned above, if A and B are in loose linkage and epistasis is recessive, we did not obtain an analytical expression for the internal equilibrium and therefore, we relied on numerical solutions to estimate p_A and p_B. The first condition corresponds to allele c invading in the background of allele b and the second one to invasion of allele c in the background of B, if B is advantageous enough.

$$
\begin{aligned}
\text{If } \beta < \;& -p_A\epsilon_{AB}(-2p_Ap_B + p_A + 2p_B) + (p_A-1)^2(p_B-1)\epsilon_{abC} \\
\gamma'_{\min} = \;& -m + p_B\left(\beta + p_A^2\epsilon_{AB} + (p_A-1)(3p_A+1)\epsilon_{abC}\right) + \epsilon_{abC} - p_A\left(2(p_A-1)p_B^2(\epsilon_{AB}+\epsilon_{abC}) + p_A\epsilon_{abC}\right) \\
\text{If } \beta > \;& p_A\epsilon_{AB}(2(p_A-1)p_B - p_A) - (p_A-1)^2(p_B-1)\epsilon_{abC} \\
\gamma'_{\min} = \;& -m - (p_B-1)(\beta + p_A\epsilon_{AB}(-2p_Ap_B + p_A + 2p_B) - 2(p_A-1)\epsilon_{abC}(p_Ap_B-1))
\end{aligned} \tag{S27}
$$

Assuming A and C are in tight linkage and B is in loose linkage, equation (S28) gives the minimal value of γ' to ensure fixation of C on the island if the epistatic interactions are

codominant. The first condition corresponds to allele c invading in the background of allele a and the second one to invasion of allele c in the background of allele A. If the epistasis between A and B is extremely strong, being associated with A comes with a cost, as c will suffer from the epistasis between A and B.

$$\begin{aligned}
\text{If } \alpha \leq \quad & \tfrac{1}{2}(-2p_B\epsilon_{AB} - p_B\epsilon_{abC} + \epsilon_{abC}), \\
& \gamma'_{\min} = -m - p_A(\alpha + p_B\epsilon_{AB}) - \tfrac{1}{2}(3p_A - 2)(p_B - 1)\epsilon_{abC} \\
\text{If } \alpha > \quad & \tfrac{1}{2}(-2p_B\epsilon_{AB} - p_B\epsilon_{abC} + \epsilon_{abC}), \\
& \gamma'_{\min} = \tfrac{1}{2}(-2m - (p_A - 1)(2\alpha + 2p_B\epsilon_{AB} + 3(p_B - 1)\epsilon_{abC})) \qquad \text{(S28)} \\
\text{With} \quad & \\
p_A = \quad & 1 - \tfrac{(\alpha-\epsilon_{abC})(\beta+\epsilon_{AB})+\sqrt{(\alpha-\epsilon_{abC})(\beta+\epsilon_{AB})((\alpha-\epsilon_{abC})(\beta+\epsilon_{AB})-4m(\epsilon_{AB}+\epsilon_{abC}))}}{2(\alpha-\epsilon_{abC})(\epsilon_{AB}+\epsilon_{abC})} \\
p_B = \quad & -\tfrac{(\alpha-\epsilon_{abC})(\beta+\epsilon_{AB})+\sqrt{(\alpha-\epsilon_{abC})(\beta+\epsilon_{AB})((\alpha-\epsilon_{abC})(\beta+\epsilon_{AB})-4m(\epsilon_{AB}+\epsilon_{abC}))}}{2(\beta+\epsilon_{AB})(\epsilon_{AB}+\epsilon_{abC})}
\end{aligned}$$

Assuming A and C are in tight linkage and B is in loose linkage, equation (S29) gives the minimal value of γ' to ensure fixation of C on the island if the epistatic interactions are recessive. As mentioned above, if $\boldsymbol{A}$ and $\boldsymbol{B}$ are in loose linkage with recessive epistasis, we do not have an analytical expression of the equilibrium and therefore, we relied on numerical solutions to estimate p_A and p_B. The first condition corresponds to allele c invading in the background of allele a and the second one to invasion of allele c in the background of allele A. If epistasis between A and B is extremely strong, being associated with A comes with a cost, as c will suffer from the epistasis between A and B.

$$\begin{aligned}
\text{If}\alpha \leq \quad & p_B\epsilon_{AB}(2p_A(p_B - 1) - p_B) - (p_A - 1)(p_B - 1)^2\epsilon_{abC} \\
& \gamma'_{\min} = -m - \alpha p_A + p_A p_B\epsilon_{AB}(2p_A(p_B - 1) - p_B) + (p_A - 1)(p_B - 1)\epsilon_{abC}((2p_A - 1)p_B - 1) \\
\text{If } \alpha > \quad & p_B\epsilon_{AB}(2p_A(p_B - 1) - p_B) - (p_A - 1)(p_B - 1)^2\epsilon_{abC} \\
& \gamma'_{\min} = -m - (p_A - 1)\left(\alpha - 2p_A(p_B - 1)p_B(\epsilon_{AB} + \epsilon_{abC}) + p_B^2\epsilon_{AB} + 2(p_B - 1)\epsilon_{abC}\right) \qquad \text{(S29)}
\end{aligned}$$

In the absence of migration, allele C is protected from invasion of allele c as long as it is advantageous on the island. This is true for all linkage architectures and dominance schemes detailed here.

In the main text, we always assume that C can "easily" fix on the island and the only condition we reported was one in which C does not contribute to local adaptation, i.e. $\gamma' > 0$ and $\beta < 0$ in the case illustrated in Fig. S9(b) and $\beta > 0$ and $-\gamma' < \text{Min}[\alpha, \beta]$ as illustrated in

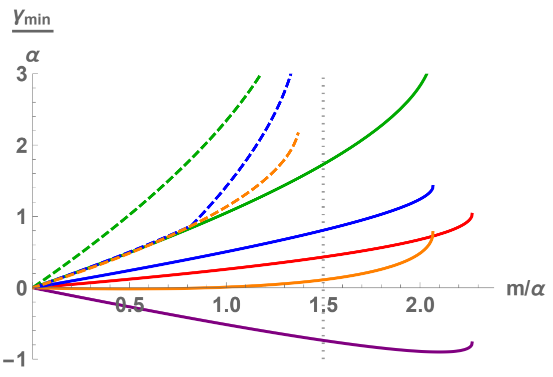

(a) $\frac{\beta}{\alpha} = -0.5$, $\frac{\epsilon_{AB}}{\alpha} = -15$, $\frac{\epsilon_{abC}}{\alpha} = -15$

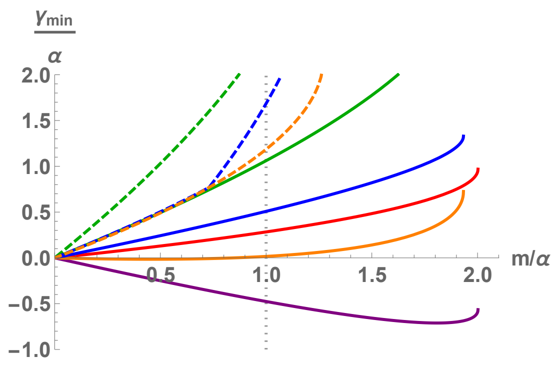

(b) $\frac{\beta}{\alpha} = 0.5$, $\frac{\epsilon_{AB}}{\alpha} = -15$, $\frac{\epsilon_{abC}}{\alpha} = -15$

Figure S9: **Fixation of C on the island, for the 3-locus epistasis model.** The X axis corresponds to the migration rate; the Y axis to $\frac{\gamma'_{\text{min}}}{\alpha}$, the minimal selective advantage of C to assure fixation of C on the island for the given migration rate. We represent this quantity for both the codominant (solid lines) and recessive cases (dashed lines) and the different linkage architectures: ***ABC*** in red, ***AB-C*** in purple, ***AC-B*** in blue, ***A-BC*** in green and ***A-B-C*** in orange. The vertical dotted black line corresponds to $\Lambda_{\text{max}}^{Ab|C}$

Fig. S9(a).

We can see here that assuming that C can "easily fix" is quite reasonable: γ' does not require a large amount of adaptation to fix on the island. At the same time, if C can fix while being deleterious, it does so only if it is slightly deleterious (and therefore we do not remove most of the state space with our restriction on $\Lambda_{\text{max}}^{Ab|C}$).

For the strength of the genetic barrier, the position of C in the genome is not important since it is fixed. Therefore, it is important to check for each scenario (***AB*** codominant, ***A-B*** codominant and ***A-B*** recessive) whether C can fix in the first place. This means that at least one element among each of the following groups ((cod.: ABC,AB-C), (cod.:AC-B, A-BC, A-B-C), (rec.:AC-B, A-BC, A-B-C)) has to allow for fixation of C for a slightly positive γ'.

As illustrated by Figure S9, migration has two opposite effects on γ_{min}. On the first hand, migration favors fixation of allele C, as migrating individuals carry this allele, therefore as migration increases, γ_{min} decreases. On the other hand, strong migration implies that haplotype ab is more frequent in the population than in a weak migration case. Therefore, the marginal fitness of allele C is reduced (due to the incompatibility being expressed), leading to γ_{min} increasing as m increases.

In Figure S9(a), $\Lambda_{\text{max}}^{Ab|C}$ is equal to 1. Therefore, Figure S9(a) illustrates that the genetic barrier can exceed the amount of local adaptation through the change of the genetic background. The C locus still does not require strong local adaptation on the island (if all loci in loose linkage (orange line), at $m = \Lambda_{\text{max}}$, $\frac{\gamma'_{\text{min}}}{\alpha} \approx 0$).

S 3.3.1 Stability of the genetic barrier with regards to the invasion of allele c

In the previous section, we have derived the minimum value of γ' that prevents allele c from invading on the island regardless of the migration rate. Alternatively, we can derive the migration rate at which the genetic barrier will collapse for a given value of $\gamma' > 0$ with regards to allele c by looking at $d(\frac{dp_C}{dt})/d(p_C)$ evaluated at the stable equilibrium (A and B polymorphic) and with $p_C = 1$. Using Mathematica, we can calculate when the genetic barrier will be lost due to allele c invading the equilibrium:

$$\begin{aligned}
\text{if} \quad & (\alpha(\beta+\epsilon_{AB})(\epsilon_{AB}+2\epsilon_{abC})+\epsilon_{AB}\epsilon_{abC}(\beta+\epsilon_{AB})+4\gamma'(\epsilon_{AB}+\epsilon_{abC})^2 < 0 \text{ or } \epsilon_{AB} < 2\epsilon_{abC}) \\
& \text{and } (\alpha(\beta+\epsilon_{AB})(\epsilon_{AB}+2\epsilon_{abC})+\epsilon_{AB}\epsilon_{abC}(\beta+\epsilon_{AB})+4\gamma'(\epsilon_{AB}+\epsilon_{abC})^2 \leq 0 \text{ or } \epsilon_{AB} \geq 2\epsilon_{abC}) \\
& \text{and } \alpha\epsilon_{AB}+2\alpha\epsilon_{abC}+\epsilon_{AB}\epsilon_{abC} > 0 \\
m < \quad & -\frac{\alpha^2\epsilon_{abC}(\beta+\epsilon_{AB})+\alpha\epsilon_{AB}(\epsilon_{abC}(\beta+\epsilon_{AB})+2\gamma'(\epsilon_{AB}+\epsilon_{abC}))-2\gamma'\epsilon_{AB}\epsilon_{abC}(\epsilon_{AB}+\epsilon_{abC})}{2\epsilon_{AB}^2(\alpha-\epsilon_{abC})} \\
& -\frac{\epsilon_{abC}|\alpha+\epsilon_{AB}|\sqrt{(\beta+\epsilon_{AB})(\alpha^2(\beta+\epsilon_{AB})+4\alpha\gamma'\epsilon_{AB}-4\gamma'\epsilon_{AB}\epsilon_{abC})}}{2\epsilon_{AB}^2(\alpha-\epsilon_{abC})}
\end{aligned} \tag{S30}$$

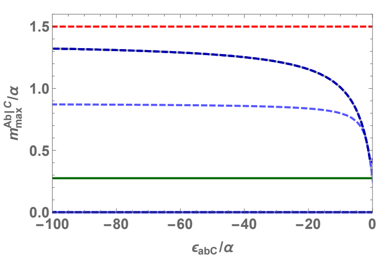

(a) $\frac{\beta}{\alpha} = -0.5$, $\frac{\epsilon_{AB}}{\alpha} = -5$

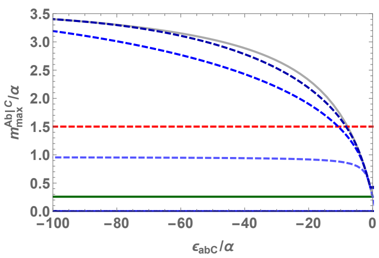

(b) $\frac{\beta}{\alpha} = -0.5$, $\frac{\epsilon_{AB}}{\alpha} = -15$

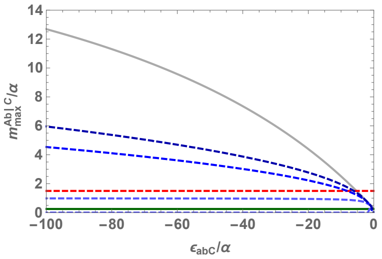

(c) $\frac{\beta}{\alpha} = -0.5$, $\frac{\epsilon_{AB}}{\alpha} = -100$

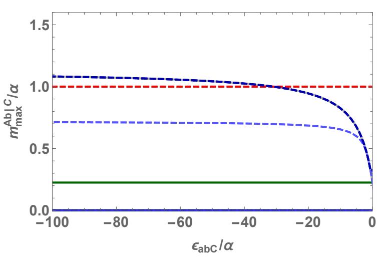

(d) $\frac{\beta}{\alpha} = 0.5$, $\frac{\epsilon_{AB}}{\alpha} = -5$

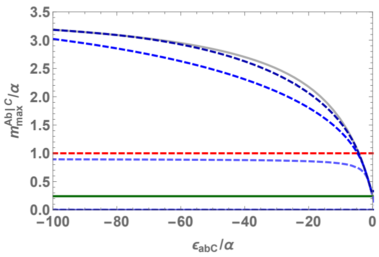

(e) $\frac{\beta}{\alpha} = 0.5$, $\frac{\epsilon_{AB}}{\alpha} = -15$

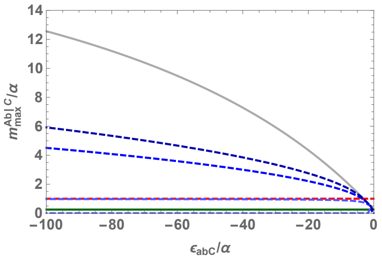

(f) $\frac{\beta}{\alpha} = 0.5$, $\frac{\epsilon_{AB}}{\alpha} = -100$

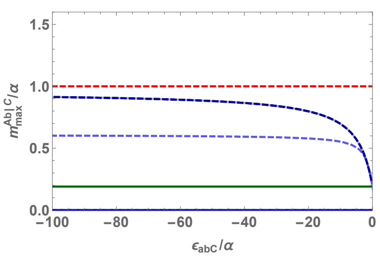

(g) $\frac{\beta}{\alpha} = 1.2$, $\frac{\epsilon_{AB}}{\alpha} = -5$

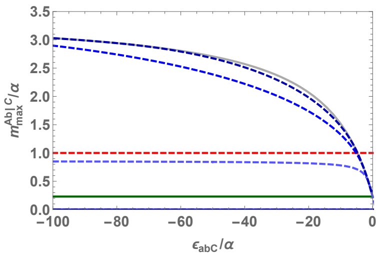

(h) $\frac{\beta}{\alpha} = 1.2$, $\frac{\epsilon_{AB}}{\alpha} = -15$

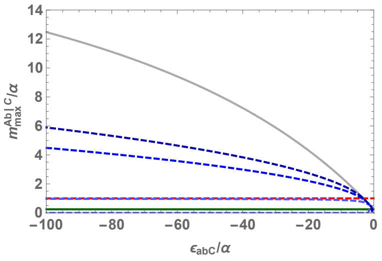

(i) $\frac{\beta}{\alpha} = 1.2$, $\frac{\epsilon_{AB}}{\alpha} = -100$

Figure S10: **Genetic barriers can still exceed $\Lambda_{\mathbf{max}}$ (red dashed line).** Here we represent the genetic barrier $m_{\max}^{Ab|C}$ derived under the assumption that allele C is advantageous enough to always remain fix on the island (gray line) and compare it to the case where allele C is only moderately advantageous (lighter blue, $\frac{\gamma'}{\alpha} = 0$, blue $\frac{\gamma'}{\alpha} = 0.2$, dark blue $\frac{\gamma'}{\alpha} = 0.4$). If allele C is moderately advantageous, it is possible that the genetic barrier is lost due to allele c being able to invade (when the blue line is below the gray line). In these cases, it remains possible to form a strong genetic barrier that will collapse due to allele c being able to invade this equilibrium (note that the final equilibrium in that case is the fixation of haplotype aBC, so invasion of allele c is temporary). If allele c is not able to invade the equilibrium, then the barrier collapses due to hybrid load.

S 3.4 Discrete model

We have seen in the main manuscript that strong incompatibilities generate strong genetic barriers to gene flow. Our results are obtained using the continuous time model, for which the equivalence between the continuous time approximation and the discrete time model is no longer automatic. Therefore, we investigated to which extent the results of the continuous time model for strong incompatibilities hold for the corresponding discrete time model. We implemented the equivalent discrete model assuming the following life cycle for haploids: reproduction (zygote formation, recombination and gametes production) followed by migration and then selection. For diploids, selection happens first, followed by migration and reproduction. These life cycles were chosen because they simplified the resulting discrete time dynamics and result in the same continuous time approximation. The observed frequencies correspond therefore to the adults for the haploid model and the genotypes of young (unselected) individuals for the diploid model. The correspondence between the selection coefficients in the discrete and continuous time models is obtained through the exponential function: for example $\alpha_d = e^{\alpha}$. Migration is obtained through the following transformation: $m_d = 1 - exp(-m)$ and loose linkage corresponds to being on two different chromosomes. Recombination between A and B is given by r_d. We used numerical simulations to determine $m_{d,\max}^{Ab|C}$ for the various cases.

For the haploid model, we explored the dynamics of the discrete time system given in equation(S31) with lethal incompatibilities, as recombination precedes selection allowing allele C to pass from the continental haplotype to an island one.

$$\begin{cases} p_A(n+1) = \frac{\alpha_d(1-m_d)p_A(n)(-r_dp_B(n)+p_A(n)+p_B(n))}{\beta_d(m_d-(m_d-1)p_B(n)(-r_dp_A(n)+p_A(n)+p_B(n)))-\alpha_d(m_d-1)p_A(n)(-r_dp_B(n)+p_A(n)+p_B(n))} \\ p_B(n+1) = \frac{\beta_d(m_d-(m_d-1)p_B(n)(-r_dp_A(n)+p_A(n)+p_B(n)))}{\beta_d(m_d-(m_d-1)p_B(n)(-r_dp_A(n)+p_A(n)+p_B(n)))-\alpha_d(m_d-1)p_A(n)(-r_dp_B(n)+p_A(n)+p_B(n))} \end{cases} \tag{S31}$$

This system is solvable and admits 3 solutions: an obvious one, fixation of the continental type and 2 potential internal equilibria. Assuming that the loci are on different chromosomes, we can derive the maximum migration rate for the formation of a stable internal equilibrium, $m_{d,\max}^{Ab|C}$, given in equation (S32).

$$m_{d,\max}^{Ab|C} = \frac{(\beta_d - 2\alpha_d)^2}{4\alpha_d^2 + 4\alpha_d\beta_d + 9\beta_d^2} \text{ if } 2\alpha_d > \beta_d \tag{S32}$$

Using equation (S32), we can derive when this genetic barrier can exceed $\Lambda_{d,\max}^{Ab|C}$. First we need to define this term in continuous time. To do so, we looked at the fitness ratio between the

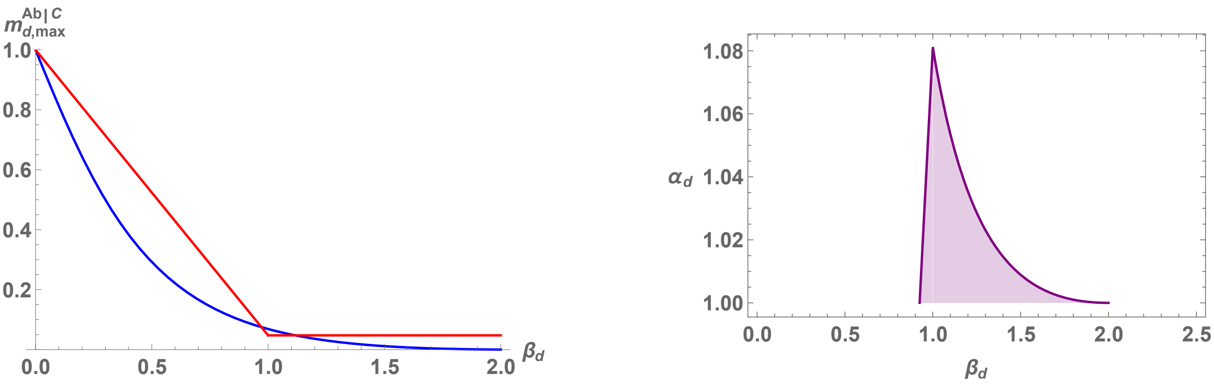

Figure S11: **Genetic barrier stronger than adaptation for the discrete model** a) We represent the genetic barrier (blue) and the maximum amount of adaptation (red) for a haploid discrete time model with lethal incompatibilities. Here we used $\alpha_d = 0.5$. b) Parameter space (purple) for which the strength of the genetic barrier can be larger than amount of local adaptation, for a haploid discrete model with lethal incompatibilities.

continental and the island haplotype. In the absence of local adaptation, this ratio is 1. If the continental type is lethal on the island, this ratio is 0. Therefore, we consider 1- the minimum of the ratio calculated along all possible evolutionary histories:

$$\Lambda_{d,\max}^{Ab|C} = 1 - \text{Min}[\frac{1}{\alpha_d}, \frac{\beta_d}{\alpha_d}] = 1 - exp(-\Lambda_{\max}^{Ab|C}) \tag{S33}$$

This also means that the transformation, between the discrete and continuous time dynamics, applied to the maximum amount of local adaptation is identical to the one applied to the maximum migration rates, which is reasonable since the quantities are comparable.

The genetic barrier can exceed the maximum amount of local adaptation if allele A is not too advantageous and B not too deleterious, see equation (S34) and Figure S11.

$$\begin{cases} \text{If } \frac{2}{\sqrt{10}-1} < \beta_d \leq 1, & \alpha_d < \frac{1}{2}\left(\sqrt{10}\beta_d - \beta_d\right) \\ \text{If } 1 < \beta_d < 2, & \alpha_d < \frac{1}{2}\left(\beta_d\sqrt{\frac{9}{2\beta_d - 1} + 1} - \beta_d\right) \end{cases} \tag{S34}$$

References

C. Bank, R. Bürger, and J. Hermisson. The Limits to Parapatric Speciation: Dobzhansky–Muller Incompatibilities in a Continent–Island Model. *Genetics*, 191(3):845–863, 2012.

A. Blanckaert and J. Hermisson. The limits to parapatric speciation II: Strengthening a preexisting genetic barrier to gene flow in parapatry. *Genetics*, 209(1):241–254, 2018.
